# Supplementary material for: Trend analysis of mortality rates and causes of death in children under 5 years old in Beijing, China from 1992 to 2015 and forecast of mortality into the future: an entire population-based epidemiological study
Source: BMJ Open. 2017 Sep 18;7(9):e015941. doi: 10.1136/bmjopen-2017-015941 (PMC5623503; doi:10.1136/bmjopen-2017-015941)
Supplement: Author's manuscript [file bmjopen-2017-015941.draft_revisions.pdf]

Trend analysis in mortality rates and causes of death for under-5 children in Beijing, China from 1992 to 2015 and forecasting mortality into the future: an entire population-based epidemiological study

Journal:	*BMJ Open*
Manuscript ID	bmjopen-2017-015941
Article Type:	Research
Date Submitted by the Author:	11-Jan-2017
Complete List of Authors:	Cao, Han; Capital Medical University School of Public Health, Epidemiology and Health Statistics; Capital Medical University School of Public Health, Beijing Key Laboratory of Clinical Epidemiology<br>Wang, Jing; Capital Medical University School of Public Health, Epidemiology and Health Statistics; Capital Medical University Beijing Obstetrics and Gynecology Hospital, Children’s Health Care<br>Li, Yichen; Capital Medical University Beijing Obstetrics and Gynecology Hospital, Children’s Health Care<br>Li, Dongyang; Capital Medical University Beijing Obstetrics and Gynecology Hospital, Children’s Health Care<br>Guo, Jin; Capital Medical University School of Public Health, Epidemiology and Health Statistics<br>Hu, Yifei; Capital Medical University School of Public Health, Child, Adolescent Health and Maternal Health<br>Meng, Kai; School of Health Administration and Education, Capital Medical University, Hospital Management<br>He, Dian; Capital Medical University, Department of Epidemiology and Biostatic, School of Public Health<br>Liu, Bin; Capital Medical University School of Public Health, Epidemiology and Health Statistics; Capital Medical University School of Public Health, Beijing Key Laboratory of Clinical Epidemiology<br>Liu, Zheng; Capital Medical University School of Public Health, Epidemiology and Health Statistics; Capital Medical University School of Public Health, Beijing Key Laboratory of Clinical Epidemiology<br>Qi, Han; Capital Medical University School of Public Health, Epidemiology and Health Statistics; Capital Medical University School of Public Health, Beijing Key Laboratory of Clinical Epidemiology<br>Zhang, Ling; Capital Medical University, Department of Epidemiology and Biostatics
**Primary Subject Heading**:	Epidemiology
Secondary Subject Heading:	Health policy, Paediatrics, Public health
Keywords:	EPIDEMIOLOGY, Health policy < HEALTH SERVICES ADMINISTRATION & MANAGEMENT, Child protection < PAEDIATRICS, Community child health < PAEDIATRICS, PUBLIC HEALTH

For peer review only - http://bmjopen.bmj.com/site/about/guidelines.xhtml

For peer review only

Trend analysis in mortality rates and causes of death for under-5 children in Beijing, China from 1992 to 2015 and forecasting mortality into the future: an entire population-based epidemiological study

Han Cao[1,3], Jing Wang[1,2], Yichen Li[2], Dongyang Li[2], Jin Guo[1], Yifei Hu[4], Kai Meng[5], Dian He[1], Bin Liu[1,3], Zheng Liu[1,3], Han Qi[1,3], Ling Zhang[1,3,*]

The first two authors contributed equally to this paper.

[*] Correspondence author

Prof. Ling Zhang, MD. Ph.D. School of Public Health, Capital Medical University, NO.10, XiToutiao YouAnmenwai, Fengtai District, Beijing, China, 100069; Tel/Fax: +86-10-83911777; E-mail: zlilyepi@ccmu.edu.cn.

Author Affiliations

1. Department of Epidemiology and Health Statistics, School of Public Health, Capital Medical University, Beijing, China.

2. Department of Children's Health Care, Beijing Obstetrics and Gynecology Hospital, Capital Medical University, Beijing, China.

3. Beijing Key Laboratory of Clinical Epidemiology, Beijing, China.

4. Department of Child, Adolescent Health and Maternal Health, School of Public Health, Capital Medical University, Beijing, China.

5. Department of Hospital Management, School of Health Administration and Education, Capital Medical University, Beijing, 100069, China.

For peer review only - http://bmjopen.bmj.com/site/about/guidelines.xhtml

Abstract

Objectives: To analyze trends in mortalities and causes of death among under-5 children in Beijing, China between 1992 and 2015, and to forecast U5MRs in the period of 2016-2020.

Methods: This is an entire population-based epidemiological study. Data was collected based on "Children's Death Reporting Card" through Beijing Under-5 mortality rate Surveillance Network. Trends in mortality rates and leading causes of death were analyzed using statistical description and chi-square test with SPSS 19.0. An ARIMA model was fitted to forecast the U5MRs between 2016 and 2020 with EViews 8.0.

Results: During 1992-2015, a total of 1,881,952 livebirths and 12,541 deaths were under surveillance. Mortality rates in neonates, infants and under-5 children had overall decreasing trends (P<0.01) from 1992 to 2015, decreasing by 84.06%, 80.04% and 80.17%. However, U5MR increased 7.20% from 2013 to 2015. Birth asphyxia, congenital heart disease, prematurity/low birth weight and other congenital abnormalities were in the top 5 causes of death during 24 years. The greatest and fastest rate reduction was pneumonia which reduced 92.26% with AARR of 10.53%. However, distribution of death causes was different in children of different ages. Accidental asphyxia and septicemia were in the top 5 of death in 28 days-1 years old children, whereas for children 1-4 years old was accidental death. U5MRs in Beijing are projected to be 2.88‰, 2.87‰, 2.90‰, 2.97‰ and 3.09‰ in the period of 2016-2020 through the predictive model.

Conclusion: Beijing has achieved great progress in reducing U5MRs from 1992 to 2015. However, U5MRs may show a slightly upward trend from 2016 to 2020 as influenced by the "Selective Two-child Policy". The future points of child health care are the management of birth asphyxia, congenital heart disease, prematurity/low birth weight and other congenital abnormalities. Importantly, specific prevention measures should be implemented for children in different age groups.

Key words: Under-5 mortality rate (U5MR); Leading causes of death; ARIMA model

For peer review only

Article Summary

Strengths and limitations in this study

▲ **Strengths**

1. Our study revealed the long-term trends and characteristics in mortality rates and leading causes of death in under-5 children through 24-year analysis.

2. We are the first to report the health condition of under-5 children after "Selective Two-child Policy" implemented in Beijing in 2014 and forecast U5MRs in the following five years.

3. The accuracy of our study is very reliable because it is an entire-population epidemiological study covering all under-5 children in all districts in Beijing.

▲ **Limitations**

1. Due to the particularity of region division in Beijing and lacking of specific data in each district, we did not analyze the differences between rural and urban areas.

2. ARIMA (1, 1, 1) model forecasts the future U5MRs reliably only for a short period of time because the model only considered the mortality varying with time instead of other possible impact factors and we have to update data continually to predict further mortality rates.

For peer review only

For peer review only - http://bmjopen.bmj.com/site/about/guidelines.xhtml

Introduction

Under-5 mortality rate (U5MR) is an important indicator which reflects children's health and the development of economy and culture in a country or area. Millennium development goal 4 (MDG4) calls for U5MR to be decreased by two-thirds between 1990 and 2015.[1] China established Children Death Surveillance and Report System in 1991 and has made great progress in reducing U5MR in past decades. The nationwide U5MR has achieved MDG4 in 2008, 7 years in advance of the target.[2-5] In 2015, U5MR in China declined to 10.7‰, a 80.1% reduction from 1990.[6] Beijing established Beijing Under-5 mortality rate Surveillance Network in 1992. However, there has been no report on the long-term dynamic trend in U5MR since the monitoring network established.

The distribution of death causes is influenced by the development of social economy and health care. There were some changes in nationwide distribution of death causes that pneumonia dropped to the third place from the first place, while congenital heart disease climbed to the fourth place from the sixth place during 1996-2006.[7] Beijing, the capital city of China, is experiencing the fastest economic and health care changes. However, the distribution of death causes in under-5 children has not been investigated in the past 24 years.

What's more, Beijing implemented "Selective Two-child Policy" in 2014[8] and "Entire Two-child Policy" in 2016[9] which has led to a boost in the number of livebirths and elder parturient.[10] It is unknown whether the policy changing will contribute to an increase in U5MR. Therefore, it is worth to establish a forecasting model to predict U5MR in advance.

The present study aimed to analyze the dynamic changes in U5MR and the distribution of death causes in Beijing, China from 1992 to 2015 and to establish a forecasting model to predict U5MR in the following five years.

Methods

Data sources

We used data from Beijing Under-5 mortality rate Surveillance Network and Beijing Maternal and Child Health Care Information System between 1992 and 2015, which were established in 1992 and 2003 respectively. Its monitoring scope covered all under-5 children in all 16 districts (Dongcheng, Xicheng, Haidian, Chaoyang, Fengtai, Shijingshan, Mentougou, Fangshan, Daxing, Shunyi, Huairou, Miyun, Yanqing, Changping, Pinggu, Tongzhou). Therefore, this is an entire population-based epidemiological study. The surveillance network was implemented rigidly according to "Health Care Operating Program for Under-5 Children in Beijing" and collected death information on the basis of "Children's Death Reporting Card" through three-level network approach (community/hospital level---districts level---municipal level).

For peer review only

For peer review only - http://bmjopen.bmj.com/site/about/guidelines.xhtml

Stringent quality control was enforced at all three levels. Maternal and child health care institutions at district level quarterly checks the “Children’s Death Reporting Card” within the jurisdiction and verify the cards with Centers for Disease Control and Prevention (CDC) at district level. What’s more, data are extracted from 20% of the communities and hospitals annually for quality control inspection and investigation of missing report. Then maternal and child health care institution at municipal level also quarterly checks the cards reported by district level and extracts data collected from 20% of the districts annually for inspection to ensure data integrity and accuracy. In addition, data-related staff of local maternal and child health care facilities are trained by specialist groups at regular intervals. Annual livebirth under reporting rate was less than 1% and death under reporting rate was less than 0.5% during recent 10 years in Beijing.

The surveillance objects are livebirths. A livebirth was defined as a fetus born after 28 gestational weeks (or with a birth weight >1000 g) with at least one of the following vital signs: heartbeat, breathing, pulsation of the umbilical cord, or contraction of voluntary muscle. Only livebirths who or whose parents have census register in Beijing were included. During 1992-2015, a total of 1,881,952 livebirths and 12,541 deaths were under surveillance. Categorization of causes of death was in accordance with the International Categorization of Diseases (ICD-10).

Procedures

We collected data based on “Children’s Death Reporting Card” of the surveillance network and information system. We used double entry and consistency check with Epidata 3.0 for data collected between 1992 and 2002; data collected between 2003 and 2015 was exported directly because electronic cards have been used since information system was established in 2003. Then, we extracted information on child’s name, gender, census register, date of birth, date of death and classification of causes of death to establish database with Excel 2016. The number of Beijing census register livebirths was exported directly from Beijing Maternal and Child Health Care Hospital.

Statistical Analysis

The trends in mortality rates and distribution of death causes were analyzed by descriptive statistics and chi-square test with SPSS 19.0. Annual average rate of reduction (AARR) was calculated as the average rate of change in mortality rates during 1992-2015, $\text{AARR}=\left(\sqrt[23]{\frac{\text{MR}_{2015}}{\text{MR}_{1992}}}-1\right)\times 100\%$.

U5MR had not only kinds of influence factors but also continuity in time. It is difficult to use traditional regression analysis methods to predict its trend. Auto regressive integrated moving average (ARIMA) model can use time to replace these kinds of influencing factors, so it is appropriate to forecast U5MR. We took U5MR from

For peer review only - http://bmjopen.bmj.com/site/about/guidelines.xhtml

1992 to 2013 as training sample to fit ARIMA model. U5MR during 1992-2013 and 2014-2015 was taken as internal and external check-up sample to do internal and external validation. The data analysis and model establishment was accomplished with EViews 8.0 and SPSS 19.0.

P<0.05 is considered significant.

Results

The Trends in Mortality Rates for Under-5 Children

The total number of death in neonates (<28 days), infants aged 28 days-1 year and children aged 1-4 years was 7619 (60.75%), 2217 (17.68%) and 2705 (21.55%) respectively. Deaths in neonates accounted for more than 50% of all deaths among for under-5 children in these 24 consecutive years.

Table 1 Mortality rates for neonates, infants and under-5 children in Beijing, China, 1992-2015

Year	Livebirths	Neonates		Infants		Under-5 years	
		Deaths	Mortality rate	Deaths	Mortality rate	Deaths	Mortality rate
1992	82105	814	9.91	1023	12.46	1278	15.57
1993	72959	661	9.06	784	10.75	1090	14.94
1994	72728	578	7.95	682	9.38	929	12.77
1995	71049	547	7.70	639	8.99	879	12.37
1996	65625	506	7.71	580	8.84	758	11.55
1997	66230	487	7.35	572	8.64	732	11.05
1998	53429	326	6.10	425	7.95	516	9.66
1999	53123	330	6.21	387	7.28	504	9.49
2000	54513	265	4.86	315	5.78	423	7.76
2001	48443	224	4.62	275	5.68	377	7.78
2002	49442	218	4.41	264	5.34	351	7.10
2003	38400	178	4.64	240	6.25	303	7.89
2004	44572	171	3.84	213	4.78	244	5.47
2005	58409	207	3.54	273	4.67	321	5.50
2006	63498	214	3.37	294	4.63	366	5.76
2007	77658	206	2.65	302	3.89	358	4.61
2008	80981	199	2.46	301	3.72	357	4.41
2009	89353	221	2.47	312	3.49	369	4.13
2010	90583	187	2.06	298	3.29	377	4.16
2011	110033	207	1.88	313	2.84	375	3.41
2012	132227	253	1.91	379	2.87	435	3.29
2013	127015	192	1.51	295	2.32	366	2.88
2014	152929	229	1.50	356	2.33	442	2.89
2015	126648	200	1.58	315	2.49	391	3.09
**AARR (%)**	-	-7.67		-6.76		-6.79	
$\chi 2$	-	3200.17		3029.94		4514.08	
P	-	<0.01		<0.01		<0.01	

For peer review only - http://bmjopen.bmj.com/site/about/guidelines.xhtml

Mortality rates are deaths in neonates, infants or under-5 children per 1000 livebirths per year.

AARR, annual average rate of reduction.

Table 1 summarizes the mortality rates for neonates (NMR), infants (IMR) and under-5 children (U5MR) in Beijing, China from 1992 to 2015. All of the mortality rates had an overall significant decreasing trend (χ^2_{NMR}=3200.17, P_{NMR}<0.01; χ^2_{IMR}=3029.94, P_{IMR}<0.01; χ^2_{U5MR}=4514.08, P_{U5MR}<0.01) and decreased by 84.06%, 80.04% and 80.17% during the study period. NMR also had the highest AARR (7.67%) from 1992 to 2015 compared to IMR (6.76%) and U5MR (6.79%). However, increases in the mortality rates were observed from 2002 to 2003 (NMR, 5.22%; IMR, 17.04%; U5MR 11.13%) and from 2014 to 2015 (NMR, 5.46%; IMR, 6.84%; U5MR 6.82%).

The Distribution of Death Causes in Under-5 Children

There were 35 species of 9 types causes of death for under-5 children under the surveillance in Beijing. The five leading causes of death were pneumonia, birth asphyxia, congenital heart disease, prematurity/low birth weight and other congenital abnormalities in 1992; while became prematurity/low birth weight, congenital heart disease, birth asphyxia, other congenital abnormalities and pneumonia in 2015. In particular, pneumonia dropped from the first place in 1992 to the fifth place in 1998, whereas, congenital heart disease climbed to the first place from the third place during the same period and has been the first cause of death for consecutive twelve years from 1998 to 2009. What's more, pneumonia dropped out of the leading five causes of death in 1999, 2003-2005 and 2007-2009; however, other neonatal diseases climbed to the fifth place in the same period.

Table 2 summarized mortality rates for leading causes of death in under-5 children, which almost had overall decreasing trends from 1992 to 2015. Deaths due to pneumonia, birth asphyxia, congenital heart disease, prematurity/low birth weight and other congenital abnormalities decreased by 92.26%, 84.56%, 78.68%, 57.18% and 77.08%. In the five leading causes of death, the fastest rate of reduction was seen in pneumonia whose AARR was 10.53%; and the slowest rate reduction was seen in prematurity/low birth weight whose AARR was only 3.62%. Particularly, prematurity/low birth weight increased continuously from 2013 to 2015, with a 44.42% growth. The similar condition could not be seen in other diseases. As for all causes of death, deaths due to neural tube defects decreased by 98.94% which had the greatest rate reduction; however, deaths due to endocrine, nutritional and metabolic disease had an increasing trend with a growth of 110.70%.

For peer review only - http://bmjopen.bmj.com/site/about/guidelines.xhtml

Table 2 Mortality rates of leading causes of death in under-5 children in Beijing, China, 1992-2015

Year	Pneumonia		Birth asphyxia		Congenital heart disease		Prematurity/low birth weight		Other congenital abnormalities		Other neonatal diseases	
	Mortality rate	Rank order	Mortality rate	Rank order	Mortality rate	Rank order	Mortality rate	Rank order	Mortality rate	Rank order	Mortality rate	Rank order
1992	295.96	1	204.62	2	181.47	3	132.76	4	120.58	5	-	-
1993	250.83	1	175.44	2	168.59	3	138.43	4	134.32	5	-	-
1994	213.12	1	140.25	4	149.87	2	141.62	3	125.12	5	-	-
1995	184.38	1	168.90	2	153.42	3	125.27	5	129.49	4	-	-
1996	144.76	2	166.10	1	143.24	3	109.71	5	121.90	4	-	-
1997	113.24	4	187.23	1	144.95	2	107.20	5	129.85	3	-	-
1998	65.51	5	131.01	2	142.24	1	95.45	4	123.53	3	-	-
1999	-	-	116.71	3	171.30	1	88.47	4	122.36	2	79.06	5
2000	51.36	5	117.40	2	170.60	1	56.87	4	95.39	3	-	-
2001	51.61	5	109.41	2	115.60	1	94.96	3	88.76	4	-	-
2002	52.59	5	97.08	2	125.40	1	86.97	3	84.95	4	-	-
2003	-	-	109.38	2	140.63	1	85.94	3	70.31	4	62.50	5
2004	-	-	65.06	4	103.20	1	87.50	2	67.31	3	47.11	5
2005	-	-	78.75	2	90.74	1	68.48	4	71.91	3	51.36	5
2006	42.52	5	72.44	2	107.09	1	58.27	4	66.14	3	-	-
2007	-	-	70.82	2	84.99	1	45.07	4	47.64	3	33.48	5
2008	-	-	56.80	2	64.21	1	38.28	4	51.86	3	34.58	5
2009	-	-	53.72	2	73.86	1	34.69	4	36.93	3	27.98	5
2010	33.12	5	38.64	3	56.30	2	61.82	1	34.22	4	-	-
2011	34.54	4	32.72	5	41.81	2	40.90	3	53.62	1	-	-
2012	22.69	5	55.21	1	38.57	3	48.40	2	34.79	4	-	-
2013	22.04	5	35.43	2	34.64	3	39.37	1	33.85	4	-	-
2014	16.35	5	26.81	4	39.23	2	54.27	1	34.66	3	-	-
2015	22.90	5	31.58	3	38.69	2	56.85	1	27.64	4	-	-
AARR (%)	-10.53		-7.80		-6.50		-3.62		-6.20		-10.28	

Mortality rates are deaths per 100000 livebirths per year.

AARR, annual average rate of reduction.

-, disease dropped out of the leading five causes of death in that year.

Figure 1 illustrates the trends in cause-specific mortality rates in different age groups from 1992 to 2015. The overall decreasing trend in mortality rates and the causes of death in under-5 children were similar to that observed in neonates (<28 days), but was different with those among 28 days-1 year and 1-4 years ch ildren, which appeared little increasing peaks in 1998, 2003, 2010 and 1993, 1999, 2006, 2010, respectively (Figure 1. C and D). The main causes of death in children among 28 days-1 year were accidental asphyxia, septicemia and other nervous system diseases besides pneumonia, congenital heart disease and other congenital abnormalities. In addition, the cause-specific mortality in congenital heart disease, other congenital abnormalities and accidental

For peer review only - http://bmjopen.bmj.com/site/about/guidelines.xhtml

asphyxia had obvious fluctuation whose largest annual rate variation were 76.19%, 90.78% and 389.90% (Figure 1C). The main causes of death in children among 1-4 years were tumor (leukemia and other tumor) and accidental death (drowning, traffic accident, accidental asphyxia, accidental poisoning, accidental fall and other accidents) besides pneumonia and congenital heart disease. The cause-specific mortality in these diseases were also fluctuated significantly, but all reduced by varying degrees compared to 1992 (Figure 1D).

The Forecasting Model for U5MR in Beijing

We used U5MR from 1992 to 2013 as the training sample to fit forecasting model through stabilization, model identification, parameter estimation and model diagnosis. U5MR from 1992 to 2013 in Beijing was a non-stationary sequence which had a decreasing trend as time goes by. After 1st difference the sequence chart showed no obvious changing trend and we confirmed it through unit root test (t=-3.05, P<0.05). Therefore, the difference order (d) was 1. From Table 3, auto correlation function (ACF) chart and partial auto correlation function (PACF) chart both showed trailing and they both decreased to 0 after lag 1. So we confirmed that p=1 and q=1. The model was confirmed to be ARIMA (1, 1, 1). The model coefficients were not zero significantly through t-test (P<0.05). R^2=0.982. Akaike information criterion (AIC) value and Bayes information criterion (BIC) value of ARIMA (1, 1, 1) model was 2.15 and 2.30, respectively. Bring the results of parameter estimation to the model general formula and the forecasting model for under-5 mortality rate was obtained:

$$x_t=-0.445+1.509x_{t-1}-0.509x_{t-2}+\alpha_t+0.999\alpha_{t-1}$$

The AC value and PAC value of residual plots of ARIMA (1, 1, 1) model were all in 95% confidence interval and P>0.05 through BOX-Ljung test. Therefore, the assumption that overall autocorrelation function was zero stood and met the white noise. So, the forecasting model we established was appropriate.

The surveillance data from 1992 to 2013 and from 2014 to 2015 was used to do model internal and external validation. Table 4 and figure 2 show the time series of predictive U5MR by the forecasting model. The actual value all fell into the 95% confidence interval and mean absolute percentage error (MAPE) was 4.76%. What's more, there was statistical significance between predictive value and actual value in neither internal validation nor external validation through Wilcoxon test ($Z_{internal}$=-0.840 , $P_{internal}$=0.401>0.05 ; $Z_{external}$=-0.447, $P_{external}$=0.655>0.05). Therefore, the internal and external prediction effect of the forecasting model were both good.

The predictive value of U5MRs from 2016 to 2020 were 2.88‰, 2.87‰, 2.90‰, 2.97‰ and 3.09‰ through the forecasting model, which indicates a slightly upward trend.

For peer review only - http://bmjopen.bmj.com/site/about/guidelines.xhtml

Table 3 ACF and PACF of 1st difference series

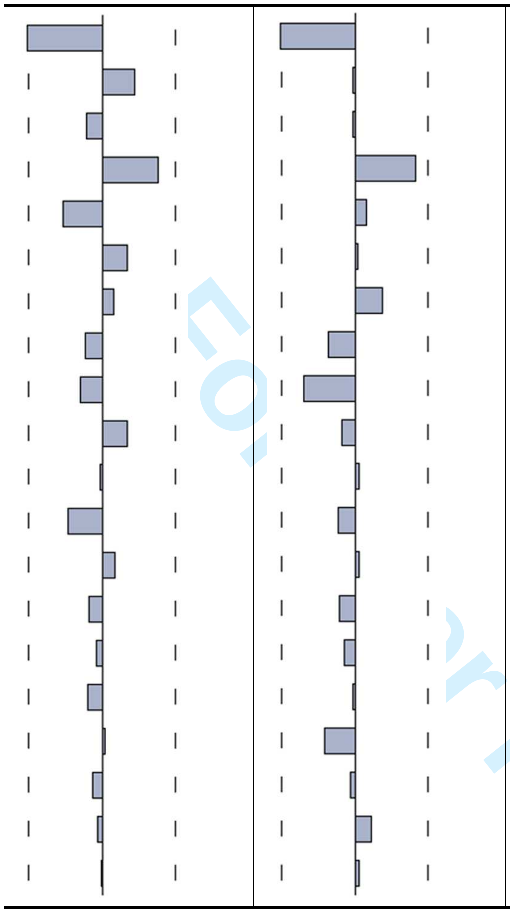

Autocorrelation	Partial Correlation		AC	PAC	*Q*-Stat	*P*
		1	-0.448	-0.448	4.840	0.028
		2	0.193	-0.009	5.788	0.055
		3	-0.094	-0.014	6.027	0.110
		4	0.336	0.363	9.239	0.055
		5	-0.235	0.068	10.913	0.053
		6	0.151	0.023	11.651	0.070
		7	0.070	0.170	11.821	0.107
		8	-0.105	-0.158	12.231	0.141
		9	-0.129	-0.306	12.897	0.167
		10	0.154	-0.082	13.932	0.176
		11	-0.011	0.032	13.938	0.236
		12	-0.199	-0.106	16.060	0.189
		13	0.080	0.031	16.449	0.226
		14	-0.079	-0.093	16.879	0.263
		15	-0.036	-0.065	16.985	0.320
		16	-0.088	-0.016	17.733	0.340
		17	0.021	-0.181	17.788	0.402
		18	-0.056	-0.029	18.300	0.436
		19	-0.025	0.106	18.451	0.493
		20	0.000	0.032	18.451	0.558

For peer review only

For peer review only - http://bmjopen.bmj.com/site/about/guidelines.xhtml

Table 4 Results of validation in ARIMA (1, 1, 1) model

Year	Actual values (‰)	Predictive values (‰)	95%CI		Relative error (%)
			Up	Down	
1992	15.57	-	-	-	-
1993	14.94	14.58	12.91	16.25	-2.41
1994	12.77	13.76	12.50	15.03	7.75
1995	12.37	12.84	11.68	14.00	3.80
1996	11.55	11.69	10.57	12.80	1.21
1997	11.05	10.82	9.73	11.91	-2.08
1998	9.66	9.98	8.91	11.05	3.31
1999	9.49	9.37	8.32	10.43	-1.26
2000	7.76	8.51	7.47	9.56	9.66
2001	7.78	8.08	7.04	9.12	3.86
2002	7.10	7.22	6.18	8.25	1.69
2003	7.89	6.65	5.62	7.68	-15.72
2004	5.47	5.80	4.77	6.82	6.03
2005	5.50	5.85	4.83	6.87	6.36
2006	5.76	5.21	4.19	6.22	-9.55
2007	4.61	4.61	3.59	5.63	0
2008	4.41	4.46	3.44	5.47	1.13
2009	4.13	4.07	3.05	5.08	-1.45
2010	4.16	3.76	2.75	4.77	-9.62
2011	3.41	3.43	2.42	4.44	0.59
2012	3.29	3.37	2.36	4.38	2.43
2013	2.88	3.17	2.16	4.17	10.07
2014	2.89	3.10	2.09	4.10	7.27
2015	3.09	2.90	1.86	3.94	-3.97

Discussion

The mortality rates for neonates, infants and under-5 children from 1992 to 2015 in Beijing, China all had an overall decreasing trend, with a reduction rate of 84.06%, 80.04% and 80.17% respectively. Particularly, neonates had the highest AARR of 7.67%. In 2011, the mortality rates for infants and children under 5 years of age decreased to 2.84‰ and 3.41‰, which met the aim (control infant mortality under 4‰ and U5MR under 5‰) of "Beijing Twelfth Five-year Period Children Development Plan (2011-2015)" four years ahead of schedule. In 2015, it further decreased to 3.09‰, the lowest among China,[11-13] which was close to the level in developed countries such as Japan (2.70‰) and Singapore (2.70‰).[14, 15] Therefore, Beijing has achieved great progress in reducing the mortality for under-5 children during the past 24 years. However, the mortality rates for neonates, infants and under-5 children all increased from 2002 to 2003 and from 2014 to 2015. The growth in 2003 was related to SARS attack in Beijing. While the growth in 2015 may be related to the policy regulation, "Selective

For peer review only

For peer review only - http://bmjopen.bmj.com/site/about/guidelines.xhtml

Two-child Policy", issued in the previous year.

Several studies have revealed an increase in mean maternal age at childbirth in Beijing since the "Selective Two-child Policy" was implemented in 2014.[10, 16] It has been known that advance maternal age was a risk factor of many diseases in under-5 children, such as prematurity and congenital abnormalities.[17-19] Our study detected that the mortality rates for neonates, infants and under-5 children increased slightly in 2015, with 5.46%, 6.84% and 6.82% growth compared to 2014 and 4.58%, 7.21% and 7.20% growth compared to 2013. What's more, the cause-specific mortality rate of prematurity/low birth weight increased 44.42% during 2013-2015. However, the cause-specific mortality rate of other diseases fluctuated in normal changing interval. Therefore, "Selective Two-child Policy" only made U5MR in Beijing occur a slightly upward trend instead of a boosting which may be the high level of health care and medical treatment reducing the influence of policy in U5MR. In this study, we predicted U5MRs between 2016 and 2020 through the forecasting model we established, 2.88‰, 2.87‰, 2.90‰, 2.97‰ and 3.09‰ respectively, which also indicated U5MR may appear a slightly upward trend in the next five years. So the maternal and child health care work in Beijing still face challenges and more investment and attention should be provided to health facilities involved into this field.

Deaths due to birth asphyxia declined steadily through Beijing intensifying intrapartum monitoring and promotion of neonatal recovery technology. Whereas, birth asphyxia was still the first and second causes of death in 2012 and 2013. There have been several studies verifying that it is closely related to increased maternal age, obesity and diabetes,[20-22] which suggests that maternal education programs targeted at the high risk groups will probably reduce the deaths from birth asphyxia further.

Congenital heart disease has been the first place of death in under-5 children in Beijing since 1998. In 2009, Beijing established a local children congenital heart disease screening and referral network. With the aim of achieving early detection, early diagnosis and early treatment, the network has improved the survival rate and living quality of children with congenital heart disease. In 2010, congenital heart disease dropped to the second cause of death. However, the cause-specific mortality of congenital heart disease in children with 28 days-1 years and 1-4 years fluctuated significantly. They even increased during some years, preventing the U5MR in Beijing from declining further. Several studies have verified that congenital heart disease has several influence factors, such as increased maternal age, prenatal infections, gestational hypertension, gestational diabetes, etc.[18, 23] Therefore, we should provide pre-pregnancy education to avoid gestational hypertension and diabetes and improve prenatal diagnosis rate to adopt measures in advance.

Prematurity/low birth weight continued to be one of the five leading causes of death in children under 5 years of

For peer review only - http://bmjopen.bmj.com/site/about/guidelines.xhtml

age in Beijing. Research in both China and the United States demonstrated that prematurity/low birth weight is related to elder parturient and multiple births by assisted reproductive technique.[12, 17, 19, 24, 25] Special prenatal care services may need to be provided to pregnant women at an advanced age and those who became pregnant using ART. Recent research suggested that higher $PM_{2.5}$ levels were associated with a higher risk of low birth weight.[26] Therefore, Beijing, with severe air pollution, should concern this point to decrease the occurrence of low birth weight.

Pneumonia had the greatest and the fastest reduction rate among the five leading causes of death which contributed to the decreasing U5MR in Beijing greatly. This may be related to the implement of neonatal visit and childhood respiratory infections prevention.[27] The remarkable decrease of mortality caused by neural tube defects was probably due to the increase intake of folic acid supplements among pregnant women in Beijing (One Hundred Achievements Promotion Plan in Ten Years in China). However, deaths due to endocrine, nutritional and metabolic disease had an increasing trend, which increased by 110.70% from 1992 to 2015. It may be relevant to elder parturient and improper feeding, but further studies and effective prevention measures are still needed.

It was worth noting that the trends in mortality rates and the composition of causes of death were significant different in children of different ages. Neonates (<28 days) have been the major group of death in under-5 children, whose proportion was more than 50%. Therefore, the entire condition in trends in mortality rates and the composition of causes of death were significantly influenced by neonates. However, the conditions in children with 28 days-1 year and 1-4 years had the obvious difference. These suggest that it is more likely to conceal the changing condition in mortality rates and the causes of death in children of different ages if we only pay attention to the overall condition in infants (including neonates and children with 28 days-1 year) and under-5 children (including neonates and children with 28 days-1 year and 1-4 years). When IMR and U5MR drop to a certain level, it may be the main causes blocking U5MR decline further, resulting a period of plateau. Therefore, for children of different ages should pay attention to different aspects and adopt different preventive measures. As for the children with 28 days-1 year, the point of prevention is accidental asphyxia through carrying on relevant knowledges and skill training to their guardians; and as for the children with 1-4 years, the point of prevention is accidental death. “Safety Beijing” action launched in 2006[28] and it is more likely to reduce the accidental death in children effectively.

Our study has several advantages. Firstly, the accuracy of this study is very reliable, because our study is an entire-population epidemiological study based on Beijing Under-5 Mortality Rate Surveillance Network and Beijing Maternal and Child Health Care System which cover all under-5 children in all districts in Beijing and

For peer review only - http://bmjopen.bmj.com/site/about/guidelines.xhtml

conquer the limitations of small samples and low coverage rate. Secondly, our study revealed the long-term trends in mortality rates and leading causes of death in under-5 children through this 24-year analysis. And we are the first to report the health condition of under-5 children after “Selective Two-child Policy” implemented in Beijing in 2014. The results of our study will provide theoretical evidence to the government in assessing the present policy and formulating further regulations related to maternal and children health care. However, several limitations should be considered when interpreting our findings. Firstly, due to the particularity of region division in Beijing and lacking of specific data in each district, we did not analyze the differences between rural and urban areas. Secondly, the ARIMA (1, 1, 1) model forecasts the future U5MRs reliably only for a short period of time because the model only considered the mortality varying with time instead of other possible impact factors, such as environment, economy and politics. We have to update data continually to predict further mortality rates.

Conclusions

Beijing has achieved great progress in reducing the mortality rates for under-5 children from 1992 to 2015. However, U5MR may show a slightly upward trend from 2016 to 2020 as influenced by the “Selective Two-child Policy”. The future points of child health care work are the management of birth asphyxia, congenital heart disease, prematurity/low birth weight and other congenital abnormalities. To further reduce the U5MR in Beijing, specific prevention measures should be adopted for children in different age groups.

Acknowledgements

Thank all healthcare workers in this surveillance system for the efforts in collecting the data.

Funding Statement

This work was supported by The National Key Research and Development Program of China grant number 2016YFC0900603.

Competing Interests

There is no competing interests in this study.

Authors’ Contribution

Han Cao: Study design, database establishment, statistical analysis and writing paper.

Jing Wang: Study design, collecting data, database establishment and writing paper.

Yichen Li and Dongyang Li: Study design and collecting data.

Jin Guo, Yifei Hu, Kai Meng, Dian He, Bin Liu, Zheng Liu, Han Qi: Database establishment.

For peer review only

Ling Zhang: Study design and direction.

References

1. WHO. Millennium Development Goals (MDGs) http://www.who.int/mediacentre/factsheets/fs290/en/2000.

2. Wang H, Liddell CA, Coates MM, et al. Global, regional, and national levels of neonatal, infant, and under-5 mortality during 1990-2013: a systematic analysis for the Global Burden of Disease Study 2013. *Lancet*. 2014;384(9947):957-79.

3. UN. The Millennium Development Goals report 2009: New York: United Nations; 2009.

4. Bryce J, Daelmans B, Dwivedi A, et al. Countdown to 2015 for maternal, newborn, and child survival: the 2008 report on tracking coverage of interventions. *Lancet*. 2008;371(9620):1247-58.

5. National Health and Family Planning Comission of the People's Republic of China. China Health Statistic Yearbook 2013. Beijing: Pecking Union Medical College Press; 2013 (in Chinese).

6. WHO. World Health Statistics 2016 data visualizations dashboard, SDG Target 3.2, Newborn and child mortality. http://apps.who.int/gho/data/view.sdg.3-2-data-ctry?lang=en2015.

7. Wang YP, Miao L, Dai L, et al. Mortality rate for children under 5 years of age in China from 1996 to 2006. *Public health*. 2011;125(5):301-7.

8. BMPC. Amendment to the Beijing population and Family Planning Ordinance. In: BMPC. http://zhengwu.beijing.gov.cn/fggz/bjdffg/t1341246.htm2014 (in Chinese).

9. BMPC. Decision of the Standing Committee of the Beijing Municipal People's Congress on Amending the regulations of Beijing Municipality on population and family planning. In: BMPC. http://www.bjrd.gov.cn/zdgz/zyfb/jyjd/201603/t20160325_161798.html2016 (in Chinese).

10. Xue Z, Kaibo L, Fengjie L, et al. A preliminary analysis of the change of high risk pregnancy with the "Selective Two-child Policy" in Beijing. *Maternal and Child Health Care of China*. 2015(24):4100-2 (in Chinese).

11. Zhang W, Chen D, Xu Y, et al. Mortality Rate for Children under 5 Years of Age in Zhejiang Province, China from 1997 to 2012. *PLoS One*. 2015;10(6):e0127770.

12. Xu H, Dai Q, Xu Y, et al. Time trends and risk factor associated with premature birth and infants deaths due to prematurity in Hubei Province, China from 2001 to 2012. *BMC pregnancy and childbirth*. 2015;15(1):329.

13. Liu YX, Jiang XM. Analysis of mortality among children under 5 years of age in Shenzhen from 2003 to 2013. *Chinese journal of contemporary pediatrics*. 2015;17(4):390-4 (in Chinese).

14. WHO. World Health Statistics 2015 http://www.who.int/gho/publications/world_health_statistics/en/2015.

15. UNICEF. State of The World's Children 2015 Country Statistical Information http://www.unicef.org/chinese/statistics/index_4733.html2015.

16. Qiaochun Q. The advantages and disadvantages of the "Selective Two-child Policy". *Population and Society*. 2014(01):3-6 (in Chinese).

17. Chen Y, Wu L, Zhang W, et al. Delivery modes and pregnancy outcomes of low birth weight infants in China. *Journal of perinatology : official journal of the California Perinatal Association*. 2016;36(1):41-6.

18. Liu X, Liu G, Wang P, et al. Prevalence of congenital heart disease and its related risk indicators among 90,796 Chinese infants aged less than 6 months in Tianjin. *International journal of epidemiology*. 2015;44(3):884-93.

19. Engle WA, Kominiarek MA. Late preterm infants, early term infants, and timing of elective deliveries. *Clinics in perinatology*. 2008;35(2):325-41.

20. Lee AC, Mullany LC, Tielsch JM, et al. Risk factors for neonatal mortality due to birth asphyxia in southern Nepal: a prospective, community-based cohort study. *Pediatrics*. 2008;121(5):e1381-90.

21. Persson M, Johansson S, Villamor E, et al. Maternal overweight and obesity and risks of severe birth-asphyxia-related complications in term infants: a population-based cohort study in Sweden. *PLoS medicine*.

For peer review only - http://bmjopen.bmj.com/site/about/guidelines.xhtml

2014;11(5):e1001648.

22. Aslam HM, Saleem S, Afzal R, et al. Risk factors of birth asphyxia. *Italian journal of pediatrics*. 2014;40:94.

23. Schulkey CE, Regmi SD, Magnan RA, et al. The maternal-age-associated risk of congenital heart disease is modifiable. *Nature*. 2015;520(7546):230-3.

24. Osterman MJ, Kochanek KD, MacDorman MF, et al. Annual summary of vital statistics: 2012-2013. *Pediatrics*. 2015;135(6):1115-25.

25. MacDorman MF, Declercq E, Zhang J. Obstetrical intervention and the singleton preterm birth rate in the United States from 1991-2006. *American journal of public health*. 2010;100(11):2241-7.

26. Fleischer NL, Merialdi M, van Donkelaar A, et al. Outdoor air pollution, preterm birth, and low birth weight: analysis of the world health organization global survey on maternal and perinatal health. *Environmental health perspectives*. 2014;122(4):425-30.

27. He SM. Clinical analysis and early prevention of neonatal pneumonia. *Guide for China Medicine*. 2011(06):60 (in Chinese).

28. Linnan H. 'Safe Beijing'project prevents child injury, disability and death in China http://www.unicef.org/infobycountry/china_34667.html: UNICEF; 2006.

For peer review only

For peer review only - http://bmjopen.bmj.com/site/about/guidelines.xhtml

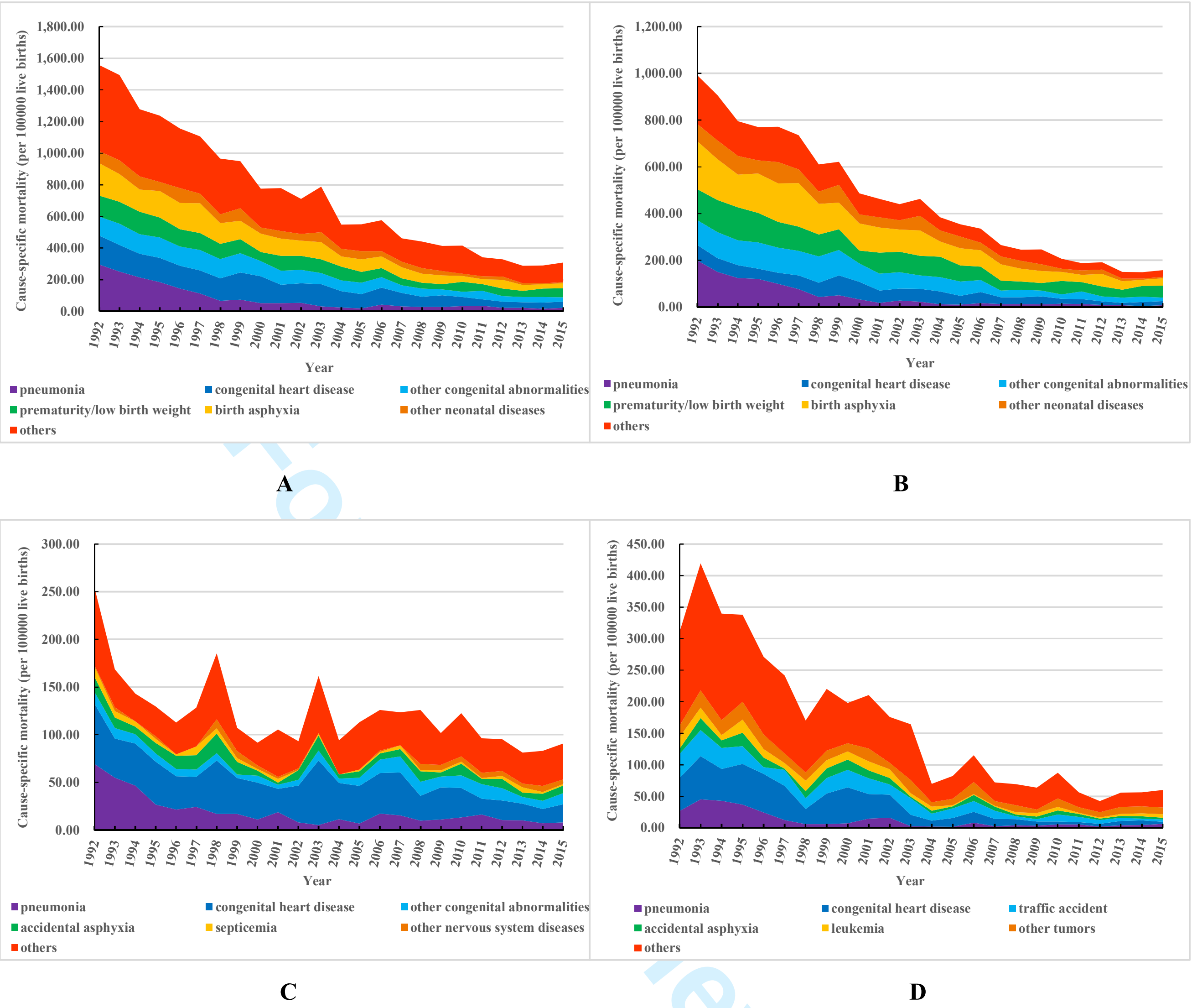

Fig. 1 The trends in cause-specific mortality rates in different ages in Beijing, China, 1992-2015: A. Under-5 children; B. Neonates (<28 days); C. 28 days-less than 1 year; D. 1-4 years.

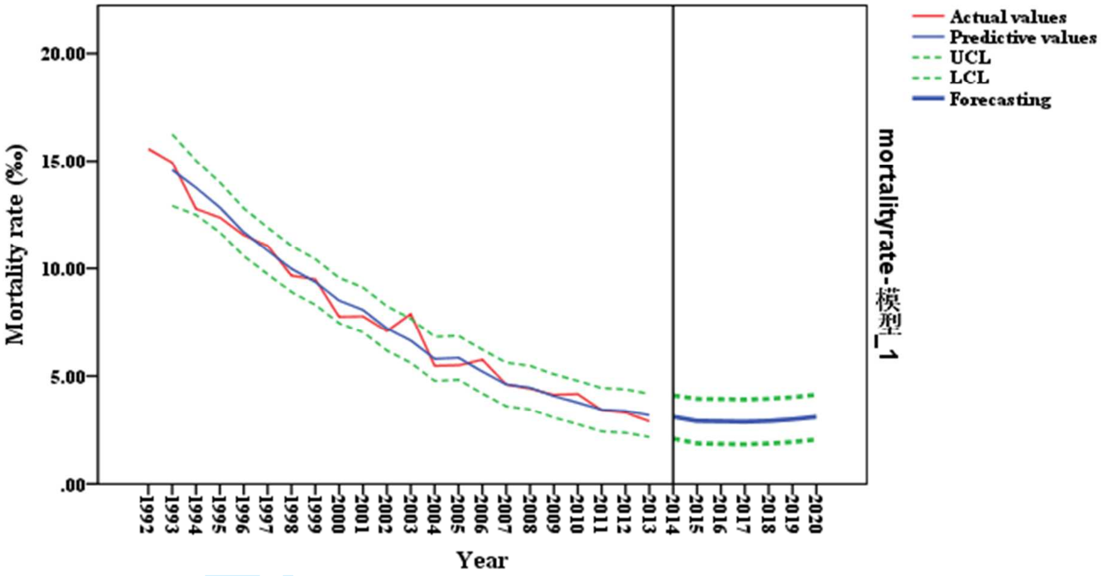

Fig. 2 Prediction of ARIMA (1, 1, 1) model

STROBE Statement

Checklist of items that should be included in reports of observational studies

Section/Topic	Item No	Recommendation	Reported on Page No
**Title and abstract**	1	(*a*) Indicate the study's design with a commonly used term in the title or the abstract	2
		(*b*) Provide in the abstract an informative and balanced summary of what was done and what was found	2
**Introduction**			
Background/rationale	2	Explain the scientific background and rationale for the investigation being reported	4
Objectives	3	State specific objectives, including any prespecified hypotheses	4
**Methods**			
Study design	4	Present key elements of study design early in the paper	4
Setting	5	Describe the setting, locations, and relevant dates, including periods of recruitment, exposure, follow-up, and data collection	4-5
Participants	6	(*a*) *Cohort study*—Give the eligibility criteria, and the sources and methods of selection of participants. Describe methods of follow-up<br>*Case-control study*—Give the eligibility criteria, and the sources and methods of case ascertainment and control selection. Give the rationale for the choice of cases and controls<br>*Cross-sectional study*—Give the eligibility criteria, and the sources and methods of selection of participants	4-5
		(*b*) *Cohort study*—For matched studies, give matching criteria and number of exposed and unexposed<br>*Case-control study*—For matched studies, give matching criteria and the number of controls per case	This is a cross-sectional study
Variables	7	Clearly define all outcomes, exposures, predictors, potential confounders, and effect modifiers. Give diagnostic criteria, if applicable	5
Data sources/measurement	8*	For each variable of interest, give sources of data and details of methods of assessment (measurement). Describe comparability of assessment methods if there is more than one group	4-5
Bias	9	Describe any efforts to address potential sources of bias	5
Study size	10	Explain how the study size was arrived at	5
Quantitative variables	11	Explain how quantitative variables were handled in the analyses. If applicable, describe which groupings were chosen and why	5-6
Statistical methods	12	(*a*) Describe all statistical methods, including those used to control for confounding	5-6
		(*b*) Describe any methods used to examine subgroups and interactions	5-6
		(*c*) Explain how missing data were addressed	5
		(*d*) *Cohort study*—If applicable, explain how loss to follow-up was addressed<br>*Case-control study*—If applicable, explain how matching of cases and controls was addressed<br>*Cross-sectional study*—If applicable, describe analytical methods taking account of sampling strategy	5
		(*e*) Describe any sensitivity analyses	6

For peer review only

For peer review only - http://bmjopen.bmj.com/site/about/guidelines.xhtml

Section/Topic	Item No	Recommendation	Reported on Page No
**Results**			
Participants	13*	(a) Report numbers of individuals at each stage of study—eg numbers potentially eligible, examined for eligibility, confirmed eligible, included in the study, completing follow-up, and analysed	6
		(b) Give reasons for non-participation at each stage	Entire population-based epidemiological study
		(c) Consider use of a flow diagram	6
Descriptive data	14*	(a) Give characteristics of study participants (eg demographic, clinical, social) and information on exposures and potential confounders	6-8
		(b) Indicate number of participants with missing data for each variable of interest	Entire population-based epidemiological study
		(c) *Cohort study*—Summarise follow-up time (eg, average and total amount)	This is a cross-sectional study
Outcome data	15*	*Cohort study*—Report numbers of outcome events or summary measures over time	This is a cross-sectional study
		Case-control study—Report numbers in each exposure category, or summary measures of exposure	This is a cross-sectional study
		Cross-sectional study—Report numbers of outcome events or summary measures	6-8
Main results	16	(*a*) Give unadjusted estimates and, if applicable, confounder-adjusted estimates and their precision (eg, 95% confidence interval). Make clear which confounders were adjusted for and why they were included	6-8
		(*b*) Report category boundaries when continuous variables were categorized	6-8
		(*c*) If relevant, consider translating estimates of relative risk into absolute risk for a meaningful time period	There is no relevant analysis in this study
Other analyses	17	Report other analyses done—eg analyses of subgroups and interactions, and sensitivity analyses	9-11
**Discussion**			
Key results	18	Summarise key results with reference to study objectives	11-13
Limitations	19	Discuss limitations of the study, taking into account sources of potential bias or imprecision. Discuss both direction and magnitude of any potential bias	14
Interpretation	20	Give a cautious overall interpretation of results considering objectives, limitations, multiplicity of analyses, results from similar studies, and other relevant evidence	14
Generalisability	21	Discuss the generalisability (external validity) of the study results	14

For peer review only - http://bmjopen.bmj.com/site/about/guidelines.xhtml

For peer review only

**Other Information**			
Funding	22	Give the source of funding and the role of the funders for the present study and, if applicable, for the original study on which the present article is based	14

**Give information separately for cases and controls in case-control studies and, if applicable, for exposed and unexposed groups in cohort and cross-sectional studies.*

Note: An Explanation and Elaboration article discusses each checklist item and gives methodological background and published examples of transparent reporting. The STROBE checklist is best used in conjunction with this article (freely available on the Web sites of PLoS Medicine at http://www.plosmedicine.org/, Annals of Internal Medicine at http://www.annals.org/, and Epidemiology at http://www.epidem.com/). Information on the STROBE Initiative is available at www.strobe-statement.org.

For peer review only

BMJ Open

Trend analysis of mortality rates and causes of death in under-5 children in Beijing, China from 1992 to 2015 and forecast of mortality into the future: an entire population-based epidemiological study

Journal:	*BMJ Open*
Manuscript ID	bmjopen-2017-015941.R1
Article Type:	Research
Date Submitted by the Author:	20-Apr-2017
Complete List of Authors:	Cao, Han; Capital Medical University School of Public Health, Epidemiology and Health Statistics; Capital Medical University School of Public Health, Beijing Key Laboratory of Clinical Epidemiology<br>Wang, Jing; Capital Medical University School of Public Health, Epidemiology and Health Statistics; Capital Medical University Beijing Obstetrics and Gynecology Hospital, Children's Health Care<br>Li, Yichen; Capital Medical University Beijing Obstetrics and Gynecology Hospital, Children's Health Care<br>Li, Dongyang; Capital Medical University Beijing Obstetrics and Gynecology Hospital, Children's Health Care<br>Guo, Jin; Capital Medical University School of Public Health, Epidemiology and Health Statistics<br>Hu, Yifei; Capital Medical University School of Public Health, Child, Adolescent Health and Maternal Health<br>Meng, Kai; School of Health Administration and Education, Capital Medical University, Hospital Management<br>He, Dian; Capital Medical University, Department of Epidemiology and Biostatic, School of Public Health<br>Liu, Bin; Capital Medical University School of Public Health, Epidemiology and Health Statistics; Capital Medical University School of Public Health, Beijing Key Laboratory of Clinical Epidemiology<br>Liu, Zheng; Capital Medical University School of Public Health, Epidemiology and Health Statistics; Capital Medical University School of Public Health, Beijing Key Laboratory of Clinical Epidemiology<br>Qi, Han; Capital Medical University School of Public Health, Epidemiology and Health Statistics; Capital Medical University School of Public Health, Beijing Key Laboratory of Clinical Epidemiology<br>Zhang, Ling; Capital Medical University, Department of Epidemiology and Biostatics
**Primary Subject Heading**:	Epidemiology
Secondary Subject Heading:	Health policy, Paediatrics, Public health
Keywords:	EPIDEMIOLOGY, Health policy < HEALTH SERVICES ADMINISTRATION & MANAGEMENT, Child protection < PAEDIATRICS, Community child health < PAEDIATRICS, PUBLIC HEALTH

For peer review only

Trend analysis of mortality rates and causes of death in under-5 children in Beijing, China from 1992 to 2015 and forecast of mortality into the future: an entire population-based epidemiological study

Han Cao[1,3], Jing Wang[1,2], Yichen Li[2], Dongyang Li[2], Jin Guo[1], Yifei Hu[4], Kai Meng[5], Dian He[1], Bin Liu[1,3], Zheng Liu[1,3], Han Qi[1,3], Ling Zhang[1,3,*]

The first two authors contributed equally to this paper.

[*] Correspondence author

Prof. Ling Zhang, MD. Ph.D. School of Public Health, Capital Medical University, No. 10, Xi Toutiao You Anmenwai, Fengtai District, Beijing, China, 100069; Tel/Fax: +86-10-83911777; E-mail: zlilyepi@ccmu.edu.cn.

Author affiliations

1. Department of Epidemiology and Health Statistics, School of Public Health, Capital Medical University, Beijing, China.

2. Department of Children's Health Care, Beijing Obstetrics and Gynecology Hospital, Capital Medical University, Beijing, China.

3. Beijing Key Laboratory of Clinical Epidemiology, Beijing, China.

4. Department of Child, Adolescent Health and Maternal Health, School of Public Health, Capital Medical University, Beijing, China.

5. Department of Hospital Management, School of Health Administration and Education, Capital Medical University, Beijing, China.

For peer review only

For peer review only - http://bmjopen.bmj.com/site/about/guidelines.xhtml

Abstract

Objectives: To analyse trends in mortality and causes of death among under-5 children in Beijing, China between 1992 and 2015, and forecast under-5 mortality rates (U5MRs) for the period 2016–2020.

Methods: An entire population-based epidemiological study was conducted. Data collection was based on the 'Child Death Reporting Card' of the Beijing Under-5 mortality rate Surveillance Network. Trends in mortality and leading causes of death were analysed using the chi-squared test and SPSS 19.0 software. An autoregressive integrated moving average (ARIMA) model was fitted to forecast U5MRs between 2016 and 2020 using the EViews 8.0 software.

Results: Mortality in neonates, infants and under-5 children decreased by 84.06%, 80.04% and 80.17% from 1992 to 2015, respectively. However, the U5MR increased by 7.20% from 2013 to 2015. Birth asphyxia, congenital heart disease, preterm/low birth weight and other congenital abnormalities comprised the top five causes of death. The greatest, most rapid reduction was that of pneumonia by 92.26%, with an annual average rate of reduction (AARR) of 10.53%. The distribution of causes of death differed among children of different ages. Accidental asphyxia and sepsis were among the top five causes of death in 28-day to 1-year-old children, as was accident in children 1–4 years old. The U5MRs in Beijing are projected to be 2.88‰, 2.87‰, 2.90‰, 2.97‰ and 3.09‰ for the period 2016–2020, based on the predictive model.

Conclusion: Beijing has made considerable progress in reducing U5MRs from 1992 to 2015. However, U5MRs could show a slight upward trend from 2016 to 2020. Future considerations for child health care include the management of birth asphyxia, congenital heart disease, prematurity/low birth weight and other congenital abnormalities. Specific preventative measures should be implemented for children of various age groups.

Key words: under-5 mortality rate; leading causes of death; autoregressive integrated moving average model

For peer review only - http://bmjopen.bmj.com/site/about/guidelines.xhtml

Article Summary

Strengths and limitations of this study

▲ **Strengths**

1. Our study revealed the long-term trends and characteristics in mortality rates and leading causes of death in under-5 children through a 24-year analysis.

2. We reported on the health conditions of under-5 children, following the implementation of the 'Selective Two-child Policy' in Beijing in 2014.

3. The accuracy of the data is reliable, because the sample comprises the entire population of all under-5 children in all districts of Beijing based on the surveillance network.

▲ **Limitations**

1. Owing to the unique regional divisions in Beijing and lack of specific data on each district, we did not analyse the differences between rural and urban areas.

2. The autoregressive integrated moving average (ARIMA) (1, 1, 1) model forecasted future U5MRs reliably for only a short period of time, because the model only considered variations in mortality with time, instead of other possible impacting factors, and data has to be continually updated to predict further mortality rates. Other model which can predict U5MRs more accuracy and capture its patterns more specific are needed to explore.

3. Because we only collected 2-year data (2014 and 2015) after the "Selective Two Child Policy", the representativeness of the changing trend and solid statistical analysis was limited. The relation between the changing trend in U5MRs and policy regulation was still unclear and needed to observe for further years. We could only give a suggestion to rise healthcare workers' attention about this potential problem.

For peer review only - http://bmjopen.bmj.com/site/about/guidelines.xhtml

Introduction

The under-5 mortality rate (U5MR) is an important indicator that reflects the health of children and development of the economy and culture of a country or region.[1] Millennium Development Goal 4 (MDG4) calls for the U5MR to be reduced by two-thirds between 1990 and 2015.[2] China established National Maternal and Child Mortality Surveillance system (MCMS) in 1996, and has made considerable progress in reducing the U5MR over past few decades.[3] With respect to the nationwide U5MR, MDG4 was achieved in 2008, seven years in advance of the target.[4] What's more, the U5MR in China declined to 10.7‰ in 2015, a 80.1% reduction from 1990.[5] Beijing established the Beijing Under-5 mortality rate Surveillance Network in 1992. However, no report on long-term dynamic trends in the U5MR has been produced since the establishment of this monitoring network.

The distribution of the causes of death is influenced by development of the social economy and health care.[6] Some changes have been noted in the nationwide distribution of the causes of death; pneumonia has dropped from the leading cause to fifth place, whereas congenital heart disease climbed from sixth place to third place from 1996–2015.[3, 6, 7] Beijing, the capital city of China, is currently experiencing the most rapid developments in the economy and health care. However, the distribution of the causes of death in under-5 children has not been investigated within the last 24 years.

Furthermore, implementation of the 'Selective Two-child Policy' in 2014[8] and the 'Entire Two-child Policy' in 2016[9] in Beijing, has led to a rise in the number of live births and elder parturients.[10] Whether or not policy changes will contribute to an increase in the U5MR remains unknown. Therefore, it is worthwhile to report the health conditions of under-5 children during this period and predict future U5MRs.

The present study aimed to analyse dynamic changes in the U5MR and the distribution of the causes of death in Beijing, China from 1992 to 2015. The study also sought to establish a forecasting model to predict the U5MR for the following five years.

Methods

Data sources

We used the surveillance data between 1992 and 2015 from the Beijing Under-5 mortality rate Surveillance Network and the Beijing Maternal and Child Health Care Information System, established in 1992 and 2003, respectively. The scope of monitoring covered all under-5 children in all 16 districts (Dongcheng, Xicheng, Haidian, Chaoyang, Fengtai, Shijingshan, Mentougou, Fangshan, Daxing, Shunyi, Huairou, Miyun, Yanqing, Changping, Pinggu, and Tongzhou). Thus, this was an entire population-based epidemiological study. The

For peer review only

surveillance network was rigidly implemented according to the 'Health Care Operating Program for Under-5 Children in Beijing' and information on deaths was collected from the 'Children's Death Reporting Cards' through a three-level network approach (community/hospital level, district level and municipal level).

Stringent quality control was enforced at all three levels. Maternal and child health care institutions at the district level checks the 'Child Death Reporting Card' within the jurisdiction on a quarterly basis and verifies the cards with the Centers for Disease Control and Prevention (CDC) at the district level. Furthermore, data from 20% of the communities and hospitals were extracted annually for quality control, inspection and investigation of missing reports. Moreover, maternal and child health care institutions at the municipal level checked the cards reported by the district on a quarterly basis, and extracted data collected annually from 20% of the districts to ensure data integrity and accuracy. In addition, data-related staff of local maternal and child health care facilities were trained by specialist groups at regular intervals. The annual live birth under-reporting rate was less than 1% and the under-reporting rate of deaths was less than 0.5% over the last 10 years in Beijing.

The main factor under surveillance was live births. A live birth was defined as a foetus born after 28 weeks of gestation (or with a birth weight >1000 g) with at least one of the following vital signs: heartbeat, breathing, pulsation of the umbilical cord, or contraction of voluntary muscle. Only live births or the parents of those births on the census register in Beijing were included. From 1992–2015, a total of 1,881,952 live births and 12,541 deaths were recorded. Categorization of the causes of death was in accordance with the International Categorization of Diseases (ICD-10).

Because the data on the deaths of under-5 children in Beijing were derived from the Beijing Under-5 Mortality Surveillance Network, no further ethical approval was required for the present study.

Procedures

We collected data from the 'Child Death Reporting Card' of the surveillance network and information system. Child Death Reporting Card was in paperboard from 1992 to 2002, so we applied double entries and consistency checks in the Epidata 3.0 software to make sure the accuracy of the data. However, data collected between 2003 and 2015 was directly exported because electronic cards have been used since the information system was established in 2003 which could ensure the accuracy of the data. We then extracted information on the child's name, gender, census register, date of birth, date of death and classification of any causes of death to establish a database in the Excel 2016 program. The number of live births recorded in the Beijing census register was exported directly from the Beijing Maternal and Child Health Care Hospital.

Statistical analysis

For peer review only - http://bmjopen.bmj.com/site/about/guidelines.xhtml

For peer review only

The trends in mortality rates and distribution of the causes of death were analysed with descriptive statistics and the chi-squared test using the SPSS 19.0 software. The neonatal mortality rate (NMR) was defined as the number of deaths within 28 days from birth per 1,000 live births. The infant mortality rate (IMR) was defined as the number of deaths within 1 year from birth per 1,000 live births. The under-5 mortality rate (U5MR) was defined as the number of deaths within 5 years from birth per 1,000 live births (as defined by the National Centre for Health Statistics). The annual average rate of reduction (AARR) was calculated as the average rate of change in mortality rates from 1992–2015 as follows:

$$AARR=\left(\sqrt[23]{\frac{MR_{2015}}{MR_{1992}}}-1\right)\times 100\%.$$

U5MR was predicted by time series analysis. The U5MR was marked by various types of influencing factors, as well as continuity in time. The use of traditional regression analysis methods to predict its trends was challenging. Time series analysis uses the factor of time to replace other kinds of influencing factors. The autoregressive integrated moving average (ARIMA) model is one of the classic methods of time series analysis, based on past values of a series and previous errors in forecasting. Therefore, it can be appropriately used to forecast the U5MR. We considered the U5MR from 1992 to 2013 as a training sample to fit the ARIMA model. The U5MRs from 1992–2013 and 2014–2015 were considered the internal and external samples for internal and external validation, respectively. Data analysis and establishment of the model was accomplished with the EViews 8.0 and SPSS 19.0 software.Statistical significance was set at $P<0.05$.

Results

Trends in mortality rates in under-5 children

The total number of deaths in neonates (<28 days), infants (<1 year), and under-5 children was 7,619 (60.75%), 9,836 (78.43%), and 12,541 (100%), respectively. Deaths in neonates accounted for more than 50% of all deaths among under-5 children during the 24 consecutive years under investigation (Figure 1).

Table 1 Mortality rates in neonates, infants and under-5 children in Beijing, China, 1992–2015

Year	Live births	Neonates		Infants		Under-5 years	
		Deaths	Mortality rate	Deaths	Mortality rate	Deaths	Mortality rate
1992	82105	814	9.91	1023	12.46	1278	15.57
1993	72959	661	9.06	784	10.75	1090	14.94
1994	72728	578	7.95	682	9.38	929	12.77
1995	71049	547	7.70	639	8.99	879	12.37
1996	65625	506	7.71	580	8.84	758	11.55
1997	66230	487	7.35	572	8.64	732	11.05
1998	53429	326	6.10	425	7.95	516	9.66

For peer review only - http://bmjopen.bmj.com/site/about/guidelines.xhtml

1999	53123	330	6.21	387	7.28	504	9.49
2000	54513	265	4.86	315	5.78	423	7.76
2001	48443	224	4.62	275	5.68	377	7.78
2002	49442	218	4.41	264	5.34	351	7.10
2003	38400	178	4.64	240	6.25	303	7.89
2004	44572	171	3.84	213	4.78	244	5.47
2005	58409	207	3.54	273	4.67	321	5.50
2006	63498	214	3.37	294	4.63	366	5.76
2007	77658	206	2.65	302	3.89	358	4.61
2008	80981	199	2.46	301	3.72	357	4.41
2009	89353	221	2.47	312	3.49	369	4.13
2010	90583	187	2.06	298	3.29	377	4.16
2011	110033	207	1.88	313	2.84	375	3.41
2012	132227	253	1.91	379	2.87	435	3.29
2013	127015	192	1.51	295	2.32	366	2.88
2014	152929	229	1.50	356	2.33	442	2.89
2015	126648	200	1.58	315	2.49	391	3.09
**AARR (%)**	-	-7.67		-6.76		-6.79	
χ2	-	3200.17		3029.94		4514.08	
P	-	<0.01		<0.01		<0.01	

NMR, neonatal mortality rate, was defined as the number of deaths within 28 days from birth per 1,000 live births.

IMR, infant mortality rate was defined as the number of deaths within 1 year from birth per 1,000 live births.

U5MR, Under-5 mortality rate was defined as the number of deaths within 5 years from birth per 1,000 live births.

AARR, annual average rate of reduction.

Table 1 summarizes the mortality rates in neonates (NMR), infants (IMR) and under-5 children (U5MR) in Beijing, China from 1992 to 2015. All mortality rates exhibited an overall trend of significant decline (χ^2_{NMR}=3200.17, P_{NMR}<0.01; χ^2_{IMR}=3029.94, P_{IMR}<0.01; χ^2_{U5MR}=4514.08, P_{U5MR}<0.01), with reductions by 84.06%, 80.04% and 80.17% during the period under investigation. The NMR had the highest AARR (7.67%) from 1992 to 2015 compared to the IMR (6.76%) and U5MR (6.79%). However, increases were observed in mortality rates from 2002 to 2003 (NMR, 5.22%; IMR, 17.04%; and U5MR 11.13%) and from 2014 to 2015 (NMR, 5.33%; IMR, 6.87%; and U5MR 6.92%).

Distribution of the leading causes of death in under-5 children

There were 35 species of 9 types causes of death for under-5 children under the surveillance in Beijing. Table 2 summarizes mortality rates for the leading causes of death in under-5 children from 1992 to 2015. The five leading causes of death were pneumonia (19.01%), birth asphyxia (13.15%), congenital heart disease (11.66%), preterm/low birth weight (8.53%) and other congenital abnormalities (7.75%) in 1992, in that order; whereas in 2015, this order became preterm/low birth weight (18.41%), congenital heart disease (12.53%), birth asphyxia (10.23%), other congenital abnormalities (8.95%) and pneumonia (7.42%). In particular, pneumonia dropped from

For peer review only - http://bmjopen.bmj.com/site/about/guidelines.xhtml

For peer review only

first place (19.01%) in 1992 to fifth place (6.78%) in 1998; whereas, congenital heart disease climbed from third place (11.66%) to first place (14.73%) during the same period, and remained the leading cause of death for 12 consecutive years from 1998 to 2009. Furthermore, pneumonia was not among the five leading causes of death during 1999, 2003–2005 and 2007–2009. However, other neonatal diseases climbed to fifth place within the same period.

The number of deaths due to pneumonia, birth asphyxia, congenital heart disease, preterm/low birth weight and other congenital abnormalities declined by 92.26%, 84.56%, 78.68%, 57.18%, and 77.08%. Among the five leading causes of death during this time period, the fastest rate of decline was observed in pneumonia, the AARR of which was 10.53%; and the slowest rate of decline was observed in preterm/low birth weight, the AARR of which was only 3.62%. In particular, preterm/low birth weight showed a steady overall increase of 44.42% from 2013 to 2015. A similar trend however, was not evident in other diseases. With respect to all causes of death, deaths due to neural tube defects showed a decline by 98.94%, which was the greatest reduction observed; however, deaths due to endocrine, nutritional and metabolic diseases showed an increasing trend by as much as 110.70%.

Table 2 Mortality rates of the leading causes of death in under-5 children in Beijing, China, 1992–2015

Year	Pneumonia		Birth asphyxia		Congenital heart disease		Preterm/low birth weight		Other congenital abnormalities		Other neonatal diseases	
	Mortality rate (Proportion)	Rank order	Mortality rate (Proportion)	Rank order	Mortality rate (Proportion)	Rank order	Mortality rate (Proportion)	Rank order	Mortality rate (Proportion)	Rank order	Mortality rate (Proportion)	Rank order
1992	295.96 (19.01)	1	204.62 (13.15)	2	181.47 (11.66)	3	132.76 (8.53)	4	120.58 (7.75)	5	76.73 (4.93)	6
1993	250.83 (16.79)	1	175.44 (11.74)	2	168.59 (11.28)	3	138.43 (9.27)	4	134.32 (8.99)	5	87.72 (5.87)	6
1994	213.12 (16.68)	1	140.25 (10.98)	4	149.87 (11.73)	2	141.62 (11.09)	3	125.12 (9.80)	5	82.50 (6.46)	6
1995	184.38 (14.90)	1	168.90 (13.65)	2	153.42 (12.40)	3	125.27 (10.13)	5	129.49 (10.47)	4	56.30 (4.55)	7
1996	144.76 (12.53)	2	166.10 (14.38)	1	143.24 (12.40)	3	109.71 (9.50)	5	121.90 (10.55)	4	92.95 (8.05)	6
1997	113.24 (10.25)	4	187.23 (16.94)	1	144.95 (13.11)	2	107.20 (9.70)	5	129.85 (11.75)	3	61.91 (5.60)	6
1998	65.51 (6.78)	5	131.01 (13.57)	2	142.24 (14.73)	1	95.45 (9.88)	4	123.53 (12.79)	3	54.28 (5.62)	6
1999	73.41 (7.74)	6	116.71 (12.30)	3	171.30 (18.06)	1	88.47 (9.33)	4	122.36 (12.90)	2	79.06 (8.33)	5
2000	51.36 (6.62)	5	117.40 (15.13)	2	170.60 (21.99)	1	56.87 (7.33)	4	95.39 (12.29)	3	38.52 (4.96)	6

For peer review only - http://bmjopen.bmj.com/site/about/guidelines.xhtml

2001	51.61 (6.63)	5	109.41 (14.06)	2	115.60 (14.85)	1	94.96 (12.20)	3	88.76 (11.41)	4	47.48 (6.10)	6
2002	52.59 (7.41)	5	97.08 (13.68)	2	125.40 (17.66)	1	86.97 (12.25)	3	84.95 (11.97)	4	42.47 (5.98)	6
2003	31.25 (3.96)	6	109.38 (13.68)	2	140.63 (17.82)	1	85.94 (10.89)	3	70.31 (8.91)	4	62.50 (7.92)	5
2004	24.68 (4.51)	6	65.06 (11.89)	4	103.20 (18.85)	1	87.50 (15.98)	2	67.31 (12.30)	3	47.11 (8.61)	5
2005	18.83 (3.43)	7	78.75 (14.33)	2	90.74 (16.51)	1	68.48 (12.46)	4	71.91 (13.08)	3	51.36 (9.35)	5
2006	42.52 (7.38)	5	72.44 (12.57)	2	107.09 (18.58)	1	58.27 (10.11)	4	66.14 (11.48)	3	33.07 (5.74)	6
2007	30.90 (6.70)	6	70.82 (15.36)	2	84.99 (18.44)	1	45.07 (9.78)	4	47.64 (10.34)	3	33.48 (7.26)	5
2008	27.17 (6.16)	6	56.80 (12.89)	2	64.21 (14.57)	1	38.28 (8.68)	4	51.86 (11.76)	3	34.58 (7.84)	5
2009	26.86 (6.50)	6	53.72 (13.01)	2	73.86 (17.89)	1	34.69 (8.40)	4	36.93 (8.94)	3	27.98 (6.78)	5
2010	33.12 (7.96)	5	38.64 (9.28)	3	56.30 (13.53)	2	61.82 (14.85)	1	34.22 (8.22)	4	14.35 (3.45)	9
2011	34.54 (10.13)	4	32.72 (9.60)	5	41.81 (12.27)	2	40.90 (12.00)	3	53.62 (15.73)	1	18.18 (5.33)	6
2012	22.69 (6.90)	5	55.21 (16.78)	1	38.57 (11.72)	3	48.40 (14.71)	2	34.79 (10.57)	4	19.66 (5.98)	6
2013	22.04 (7.65)	5	35.43 (12.30)	2	34.64 (12.02)	3	39.37 (13.66)	1	33.85 (11.75)	4	11.81 (4.10)	8
2014	16.35 (5.66)	5	26.81 (9.28)	4	39.23 (13.57)	2	54.27 (18.78)	1	34.66 (11.99)	3	5.23 (1.81)	14
2015	22.90 (7.42)	5	31.58 (10.23)	3	38.69 (12.53)	2	56.85 (18.41)	1	27.64 (8.95)	4	6.32 (2.05)	13
AARR (%)	-10.53		-7.80		-6.50		-3.62		-6.20		-10.28	

Mortality rates are presented as deaths per 100000 live births per year.

Proportion, %.

AARR, annual average rate of reduction.

Figure 2 illustrates the trends in cause-specific mortality rates among different age groups (children aged <28 days; 28 days to 1 year; and 1–4 years) from 1992 to 2015. The overall trend of decline in mortality rates and the causes of death in under-5 children were similar to those observed in neonates (<28 days) (Figure 2. A and B), but different from those among the age groups 28 days to 1 year and 1–4 years, which exhibited slightly increasing peaks in 1998, 2003 and 2010, and 1993, 1999, 2006 and 2010, respectively (Figure 2. C and D). The main causes of death among children 28 days to 1 year were accidental asphyxia, sepsis and other neurological disease, in

For peer review only
For peer review only - http://bmjopen.bmj.com/site/about/guidelines.xhtml

addition to pneumonia, congenital heart disease, and other congenital abnormalities. Furthermore, the cause-specific mortality rates due to congenital heart disease, other congenital abnormalities, and accidental asphyxia showed obvious fluctuations with the largest annual variations of 77.08% (from 31.71 in 1997 to 56.15 in 1998 per 100,000 livebirths), 94.90% (from 3.76 in 1999 to 7.34 in 2000 per 100,000 livebirths) and 389.90% (from 2.06 in 2001 to 10.11 in 2002 per 100,000 livebirths) (Figure 2C). The main causes of death in children 1–4 years were tumours (leukaemia and other tumours) and accidents (drowning, traffic accident, accidental asphyxia, accidental poisoning, accidental fall, and other accidents) in addition to pneumonia and congenital heart disease. The cause-specific mortality in these diseases also showed significant fluctuations, but were all reduced by varying degrees after 1992 (Figure 2D).

Forecasting model for the U5MR in Beijing

We used the U5MR from 1992 to 2013 as the training sample to fit the forecasting model through stabilization, model identification, parameter estimation and model diagnosis. The U5MR in Beijing from 1992 to 2013 showed a non-stationary declining trend with time. After the first notable difference, the sequence chart showed no obvious changes in trend, as confirmed by the unit root test (t=-3.05, P<0.05). Therefore, the difference in order (d) was 1. From Table 3, the auto correlation function (ACF) and partial autocorrelation function (PACF) charts both showed trailing and declined to 0 after lag 1. Thus, we confirmed that p=1 and q=1. The model was confirmed to be ARIMA (1, 1, 1). The model coefficients were not significantly zero according to the t-test (P<0.05, R^2=0.982). The Akaike information criterion (AIC) and Bayes information criterion (BIC) values of the ARIMA (1, 1, 1) model were 2.15 and 2.30, respectively. The results of parameter estimation were incorporated with the general formula of the model to obtain the forecasting model for the under-5 mortality rate as follows:

$$x_t=-0.445+1.509x_{t-1}-0.509x_{t-2}+\alpha_t+0.999\alpha_{t-1}$$

The autocorrelation (AC) and partial autocorrelation (PAC) values of the residual plots of the ARIMA (1, 1, 1) model were all within the 95% confidence interval (P>0.05) based on the Box–Ljung test. Therefore, the assumption that the overall autocorrelation function was zero stood and met the criteria for white noise. Thus, the forecasting model we established was appropriate.

The surveillance data from 1992–2013 and 2014–2015 was used for internal and external validation of the model. Table 4 and Figure 3 show the time series of the predictive U5MR based on the forecasting model. The actual values all fell within the 95% confidence interval and the mean absolute percentage error (MAPE) was 4.76%. Furthermore, statistically significant differences between the predictive and actual values were not observed in either the internal or external validation, based on the results of the Wilcoxon test ($Z_{internal}$=-0.840,

For peer review only - http://bmjopen.bmj.com/site/about/guidelines.xhtml

$P_{internal}$=0.401>0.05; $Z_{external}$=-0.447, $P_{external}$=0.655>0.05). Therefore, the internal and external prediction effects of the forecasting model were both favourable.

The predictive value of the U5MRs from 2016–2020 were 2.88‰, 2.87‰, 2.90‰, 2.97‰ and 3.09‰ based on the forecasting model, which indicated a slight upward trend.

Table 3 Auto correlation function and partial autocorrelation functions of the first difference time series

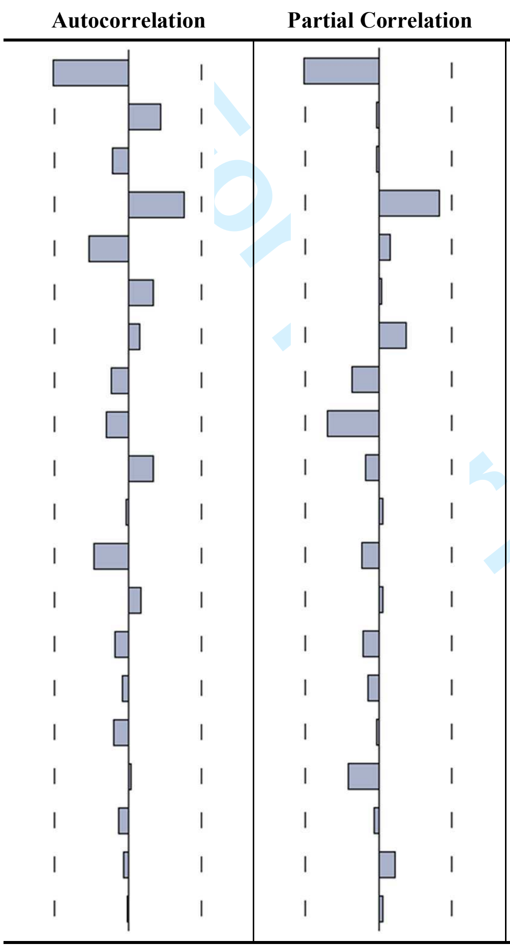

	AC	PAC	*Q*-Stat	*P*
1	-0.448	-0.448	4.840	0.028
2	0.193	-0.009	5.788	0.055
3	-0.094	-0.014	6.027	0.110
4	0.336	0.363	9.239	0.055
5	-0.235	0.068	10.913	0.053
6	0.151	0.023	11.651	0.070
7	0.070	0.170	11.821	0.107
8	-0.105	-0.158	12.231	0.141
9	-0.129	-0.306	12.897	0.167
10	0.154	-0.082	13.932	0.176
11	-0.011	0.032	13.938	0.236
12	-0.199	-0.106	16.060	0.189
13	0.080	0.031	16.449	0.226
14	-0.079	-0.093	16.879	0.263
15	-0.036	-0.065	16.985	0.320
16	-0.088	-0.016	17.733	0.340
17	0.021	-0.181	17.788	0.402
18	-0.056	-0.029	18.300	0.436
19	-0.025	0.106	18.451	0.493
20	0.000	0.032	18.451	0.558

AC, autocorrelation; PAC, partial correlation, Q-Stat, Q-statistic

For peer review only - http://bmjopen.bmj.com/site/about/guidelines.xhtml

Table 4 Results of validation in the ARIMA (1, 1, 1) model

Year	Actual values (‰)	Predictive values (‰)	95% CI		Relative error (%)
			Upper	Lower	
1992	15.57	-	-	-	-
1993	14.94	14.58	12.91	16.25	-2.41
1994	12.77	13.76	12.50	15.03	7.75
1995	12.37	12.84	11.68	14.00	3.80
1996	11.55	11.69	10.57	12.80	1.21
1997	11.05	10.82	9.73	11.91	-2.08
1998	9.66	9.98	8.91	11.05	3.31
1999	9.49	9.37	8.32	10.43	-1.26
2000	7.76	8.51	7.47	9.56	9.66
2001	7.78	8.08	7.04	9.12	3.86
2002	7.10	7.22	6.18	8.25	1.69
2003	7.89	6.65	5.62	7.68	-15.72
2004	5.47	5.80	4.77	6.82	6.03
2005	5.50	5.85	4.83	6.87	6.36
2006	5.76	5.21	4.19	6.22	-9.55
2007	4.61	4.61	3.59	5.63	0
2008	4.41	4.46	3.44	5.47	1.13
2009	4.13	4.07	3.05	5.08	-1.45
2010	4.16	3.76	2.75	4.77	-9.62
2011	3.41	3.43	2.42	4.44	0.59
2012	3.29	3.37	2.36	4.38	2.43
2013	2.88	3.17	2.16	4.17	10.07
2014	2.89	3.10	2.09	4.10	7.27
2015	3.09	2.90	1.86	3.94	-3.97

Discussion

The mortality rates in neonates, infants and under-5 children from 1992 to 2015 in Beijing, China all showed an overall trend of decline, with reductions by 84.06%, 80.04% and 80.17%, respectively. Neonates in particular, had the highest AARR of 7.67%. In 2011, the mortality rates in infants and under-5 children was reduced to 2.84‰ and 3.41‰, respectively, thereby achieving one of the aims (reduce infant mortality to <4‰ and the U5MR to <5‰) of the 'Beijing Twelfth Five-year Period Children Development Plan (2011–2015)' four years ahead of schedule. In 2015, the U5MR was further reduced to 3.09‰, the lowest in China,[11-13] which was close to the levels achieved in developed countries, such as Japan (2.70‰) and Singapore (2.70‰).[14, 15] Thus, Beijing has made considerable progress in reducing mortality of under-5 children over the past 24 years. Nevertheless, the mortality rates in neonates, infants and under-5 children all increased from 2002–2003 and 2014–2015. The increase in 2003 was related to the severe acute respiratory syndrome (SARS) outbreak in Beijing. Whereas the

For peer review only - http://bmjopen.bmj.com/site/about/guidelines.xhtml

For peer review only

increase in 2015 might have been related to the 'Selective Two-child Policy' regulation, issued in the previous year.

Several studies have reported an increase in the mean maternal age at childbirth in Beijing since the 'Selective Two-child Policy' was implemented in 2014.[10, 16] Advanced maternal age has been a proven risk factor of many disorders in under-5 children, such as prematurity and congenital abnormalities.[17-19] In the present study, the mortality rates in neonates, infants and under-5 children increased slightly in 2015, by 5.33%, 6.87% and 6.92% compared to 2014. Furthermore, the cause-specific mortality rate of preterm/low birth weight increased by 44.42% during the period 2013–2015. However, the cause-specific mortality rate of other diseases fluctuated with regular changes in interval. Therefore, we suspected that the 'Selective Two-child Policy' may induce only a slight upward trend in the U5MR in Beijing instead of a major boost. This finding could be attributed mainly to the high level of health care and medical treatment available and is less likely caused by the influence of the policy on the U5MR. Using the forecasting model established in the present study, we predicted the U5MRs from 2016 to 2020, to be 2.88‰, 2.87‰, 2.90‰, 2.97‰, and 3.09‰, respectively, indicating a slight upward trend over the next five years. Thus, our suggestion is that maternal and child health care in Beijing still faces challenges and greater investment and attention should be provided to health facilities involved in this field.

Deaths due to birth asphyxia decreased by 84.56% from 1992 to 2015 in Beijing. On the one hand, it benefited from intensification of intrapartum monitoring and promotion of neonatal recovery technology in Beijing. On the other hand, it may be related to the increase of caesarean. There have been several studies verifying that the increase of caesarean section rate can reduce the occurrence of birth asphyxia.[20, 21] There has been a sharp rise in caesarean section rate in Beijing since 1999 and it reached 57% during 2009 and 2013,[22] which may take part in reducing the deaths in birth asphyxia. Whereas birth asphyxia remained the first and second causes of death in 2012 and 2013, respectively. Increased maternal age, obesity and diabetes are the risk factors of birth asphyxia,[23-25] suggesting that maternal education programs targeted at high-risk groups will probably further reduce the number of deaths due to birth asphyxia.

Congenital heart disease has been the number one cause of death in under-5 children in Beijing since 1998. In 2009, Beijing established a local children's congenital heart disease screening and referral network. With the aim of achieving early detection, diagnosis, and treatment, the network has improved the survival rate and quality of life for children with congenital heart disease. In 2010, congenital heart disease dropped to become the second leading cause of death. However, the cause-specific mortality of congenital heart disease in children aged 28 days to 1 year and 1–4 years showed significant fluctuations. These values increased during some years, thus

For peer review only - http://bmjopen.bmj.com/site/about/guidelines.xhtml

preventing the U5MR in Beijing from declining further. The maternal age in Beijing is increasing yearly and elderly parturient accounted for 20.04% in 2014.[10] What's more, the increased maternal age is closely related to the occurrence of gestational hypertension and gestational diabetes and they are all the risk factors of congenital heart disease.[18, 26] Therefore, we should advocate pregnancy in appropriate age and improve prenatal diagnosis to decrease the occurrence of congenital heart disease.

Preterm/low birth weight continued to be one of the five leading causes of death in under-5 children in Beijing. This may be explained by the increasing caesarean section rate in Beijing. The elder parturient and multiple births by assisted reproductive technique both promoted the rate of caesarean section which has reached 57% in 2014 in Beijing.[22] Special prenatal care services might need to be provided to pregnant women of an advanced age and those who became pregnant using assisted reproductive technologies. Recent research suggests that higher levels of particulate matter of diameter <2.5 μm ($PM_{2.5}$) were associated with a higher risk of low birth weight.[27] Therefore, given the levels of severe air pollution in Beijing, this should be a major point of concern to reduce the incidence of low birth weight.

Pneumonia showed the greatest and fastest rate of reduction among the five leading causes of death, and this contributed considerably to the reduced U5MR in Beijing. This might be related to the implementation of neonatal visits and the prevention of childhood respiratory infections.[28] The remarkable reduction in mortality caused by neural tube defects was probably due to the increased intake of folic acid supplements among pregnant women in Beijing (One Hundred Achievements Promotion Plan in Ten Years in China). However, deaths due to endocrine, nutritional and metabolic disease showed a general trend of increase by 110.70%, from 1992 to 2015. This might be related to elder parturients, and poor dietary practices; however, further studies and effective preventative measures are still required.

It is worth noting that trends in mortality rates and the composition of the causes of death were significantly different in children of different ages. Neonatal mortality (<28 days) accounted for more than 50% of deaths in under-5 children. Therefore, the general trends in mortality and specific of causes of death were significantly influenced by neonates. Nevertheless, the conditions in children aged 28 days to 1 year and 1–4 years showed obvious differences. Therefore, different aspects should receive greater attention among the various age groups, so that different preventative measures can be adopted for children of different ages. For children aged 28 days to 1 year, the key focus of preventative measures should be accidental asphyxia through training of the guardians in the relevant knowledge and skills. For children aged 1–4 years, the key focus of preventative measures should be accidental death. The 'Safety Beijing' action plan launched in 2006[29] is likely to effectively reduce the number of

For peer review only - http://bmjopen.bmj.com/site/about/guidelines.xhtml

accident in children.

Our study has several advantages. Firstly, the data is reliable; because our study considers the entire population, based on the Beijing Under-5 Mortality Rate Surveillance Network and Beijing Maternal and Child Health Care System, which covers all under-5 children in all districts of Beijing. Thus, the present study is not limited by a small sample size, or low coverage rate. Secondly, our study revealed the long-term trends in mortality rates and leading causes of death in under-5 children through a 24-year analysis. Furthermore, we reported the health conditions of under-5 children following the implementation of the 'Selective Two-child Policy' in Beijing in 2014. The results of our study will provide suggestion for the government to assess the present policy and formulate further regulations related to maternal and child health care. However, several limitations should be considered when interpreting our findings. Firstly, because of the unique regional divisions in Beijing and the lack of specific data on each district, we did not analyse the differences between rural and urban areas. Secondly, the ARIMA (1, 1, 1) model forecasts future U5MRs reliably, but only for a short period. This is because the model only considered variations in mortality with time, instead of other possible impacting factors, such as environment, economy and politics. Data must be continually updated to predict further mortality rates. Other model which can predict U5MRs more accuracy and capture its patterns more specific are needed to explore. Thirdly, since we only collected 2-year data (2014 and 2015) after the "Selective Two Child Policy" (2014), the representativeness of the changing trend and solid statistical analysis was limited. The relation between the changing trend in U5MRs and policy regulation was still unclear and needed to observe for further years. We could only give a suggestion to rise healthcare workers' attention about this potential problem.

Conclusions

From 1992 to 2015, Beijing has made considerable progress in reducing the mortality rates of under-5 children. However, the U5MR might show a slight upward trend from 2016 to 2020. The key points for future consideration regarding child health care include the management of birth asphyxia, congenital heart disease, prematurity/low birth weight and other congenital abnormalities. To further reduce the U5MR in Beijing, specific prevention measures should be adopted for children of various age groups.

Acknowledgements

We thank all healthcare workers in the surveillance system for their efforts in data collection.

Funding Statement

This work was supported by The National Key Research and Development Program of China grant number

For peer review only - http://bmjopen.bmj.com/site/about/guidelines.xhtml

2016YFC0900603.

Competing Interests

There are no competing interests to declare.

Data Sharing Statement

No additional data are available.

Authors' Contribution

Han Cao: study design, database establishment, statistical analysis and writing of the paper.

Jing Wang: study design, data collection, database establishment and writing of the paper.

Yichen Li and Dongyang Li: study design and data collection.

Jin Guo, Yifei Hu, Kai Meng, Dian He, Bin Liu, Zheng Liu, Han Qi: database establishment.

Ling Zhang: study design, direction of statistical analysis and writing and modification of the paper.

References

1. Chengye J. Child and Adolescent Health. Beijing: People's Medical Publishing House; 2012 (in Chinese).
2. WHO. Millennium Development Goals (MDGs) http://www.who.int/mediacentre/factsheets/fs290/en/2000.
3. He C, Liu L, Chu Y, et al. National and subnational all-cause and cause-specific child mortality in China, 1996-2015: a systematic analysis with implications for the Sustainable Development Goals. *The Lancet Global health*. 2017;5(2):186-97.
4. Wang H, Liddell CA, Coates MM, et al. Global, regional, and national levels of neonatal, infant, and under-5 mortality during 1990-2013: a systematic analysis for the Global Burden of Disease Study 2013. *Lancet*. 2014;384(9947):957-79.
5. WHO. World Health Statistics 2016 data visualizations dashboard, SDG Target 3.2, Newborn and child mortality http://apps.who.int/gho/data/view.sdg.3-2-data-ctry?lang=en2015.
6. Song P, Theodoratou E, Li X, et al. Causes of death in children younger than five years in China in 2015: an updated analysis. *Journal of global health*. 2016;6(2):020802.
7. Wang YP, Miao L, Dai L, et al. Mortality rate for children under 5 years of age in China from 1996 to 2006. *Public health*. 2011;125(5):301-7.
8. BMPC. Amendment to the Beijing population and Family Planning Ordinance. In: (BMPC). http://zhengwu.beijing.gov.cn/fggz/bjdffg/t1341246.htm2014 (in Chinese).
9. BMPC. Decision of the Standing Committee of the Beijing Municipal People's Congress on Amending the regulations of Beijing Municipality on population and family planning. In: (BMPC). http://www.bjrd.gov.cn/zdgz/zyfb/jyjd/201603/t20160325_161798.html2016 (in Chinese).
10. Xue Z, Kaibo L, Fengjie L, et al. A preliminary analysis of the change of high risk pregnancy with the "Selective Two-child Policy" in Beijing. *Maternal and Child Health Care of China*. 2015(24):4100-2 (in Chinese).
11. Zhang W, Chen D, Xu Y, et al. Mortality Rate for Children under 5 Years of Age in Zhejiang Province, China from 1997 to 2012. *PLoS One*. 2015;10(6):e0127770.
12. Xu H, Dai Q, Xu Y, et al. Time trends and risk factor associated with premature birth and infants deaths due to

For peer review only

For peer review only - http://bmjopen.bmj.com/site/about/guidelines.xhtml

prematurity in Hubei Province, China from 2001 to 2012. *BMC pregnancy and childbirth*. 2015;15(1):329.

13. Liu YX, Jiang XM. Analysis of mortality among children under 5 years of age in Shenzhen from 2003 to 2013. *Chinese journal of contemporary pediatrics*. 2015;17(4):390-4 (in Chinese).

14. WHO. World Health Statistics 2015 http://www.who.int/gho/publications/world_health_statistics/en/2015.

15. UNICEF. State of The World's Children 2015 Country Statistical Information http://www.unicef.org/chinese/statistics/index_4733.html2015.

16. Qiaochun Q. The advantages and disadvantages of the "Selective Two-child Policy". *Population and Society*. 2014(01):3-6 (in Chinese).

17. Chen Y, Wu L, Zhang W, et al. Delivery modes and pregnancy outcomes of low birth weight infants in China. *Journal of perinatology : official journal of the California Perinatal Association*. 2016;36(1):41-6.

18. Liu X, Liu G, Wang P, et al. Prevalence of congenital heart disease and its related risk indicators among 90,796 Chinese infants aged less than 6 months in Tianjin. *International journal of epidemiology*. 2015;44(3):884-93.

19. Engle WA, Kominiarek MA. Late preterm infants, early term infants, and timing of elective deliveries. *Clinics in perinatology*. 2008;35(2):325-41.

20. Ramachandrappa A, Jain L. Elective cesarean section: its impact on neonatal respiratory outcome. *Clinics in perinatology*. 2008;35(2):373-93.

21. Coutinho PR, Cecatti JG, Surita FG, et al. Perinatal outcomes associated with low birth weight in a historical cohort. *Reproductive health*. 2011;8:18.

22. Ruiya Q, Zhenghong C, Weiyuan Z, et al. The changing trend in cesarean section rate and indication analysis in Beijing area. *Journal of Medical Research*. 2016(12):112-6 (in Chinese).

23. Lee AC, Mullany LC, Tielsch JM, et al. Risk factors for neonatal mortality due to birth asphyxia in southern Nepal: a prospective, community-based cohort study. *Pediatrics*. 2008;121(5):1381-90.

24. Persson M, Johansson S, Villamor E, et al. Maternal overweight and obesity and risks of severe birth-asphyxia-related complications in term infants: a population-based cohort study in Sweden. *PLoS medicine*. 2014;11(5):e1001648.

25. Aslam HM, Saleem S, Afzal R, et al. Risk factors of birth asphyxia. *Italian journal of pediatrics*. 2014;40:94.

26. Schulkey CE, Regmi SD, Magnan RA, et al. The maternal-age-associated risk of congenital heart disease is modifiable. *Nature*. 2015;520(7546):230-3.

27. Fleischer NL, Merialdi M, van Donkelaar A, et al. Outdoor air pollution, preterm birth, and low birth weight: analysis of the world health organization global survey on maternal and perinatal health. *Environmental health perspectives*. 2014;122(4):425-30.

28. He SM. Clinical analysis and early prevention of neonatal pneumonia. *Guide for China Medicine*. 2011(06):60 (in Chinese).

29. Linnan H. 'Safe Beijing'project prevents child injury, disability and death in China http://www.unicef.org/infobycountry/china_34667.html: UNICEF; 2006.

Figure Legends

Fig 1. Age composition of the death in under-5 children in Beijing, China, 1992-2015.

Fig 2. Trends in cause-specific mortality rates among different ages in Beijing, China, 1992–2015: A. under-5 children; B. neonates (<28 days); C. 28 days to <1 year; D. 1–4 years.

Fig 3. Prediction of ARIMA (1, 1, 1) model (UCL, upper control limit; LCL, lower control limit).

For peer review only

For peer review only - http://bmjopen.bmj.com/site/about/guidelines.xhtml

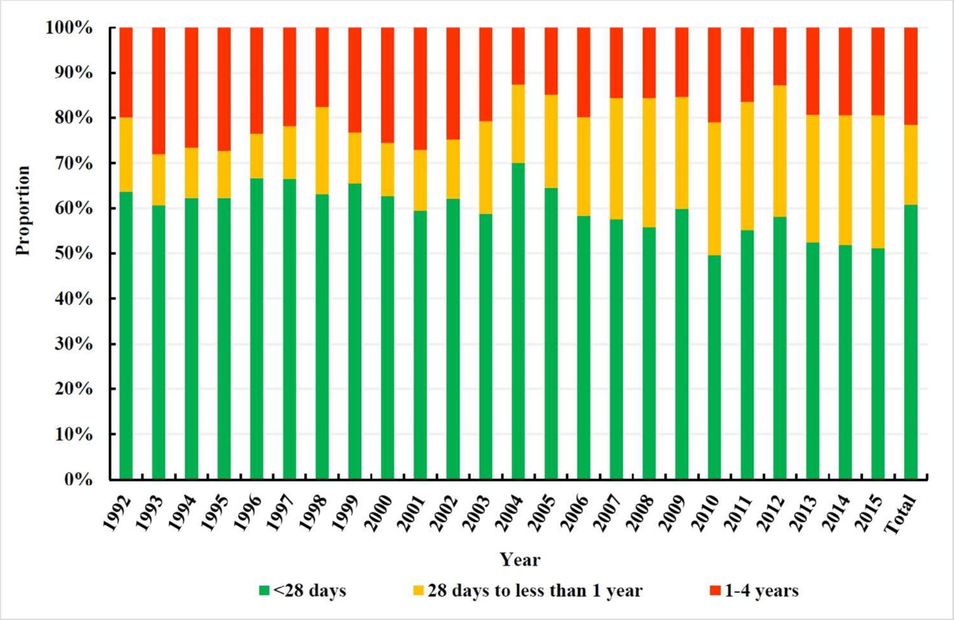

Fig. 1 Age composition of the death in under-5 children in Beijing, China, 1992-2015.

Fig 1. Age composition of the death in under-5 children in Beijing, China, 1992-2015.

133x94mm (300 x 300 DPI)

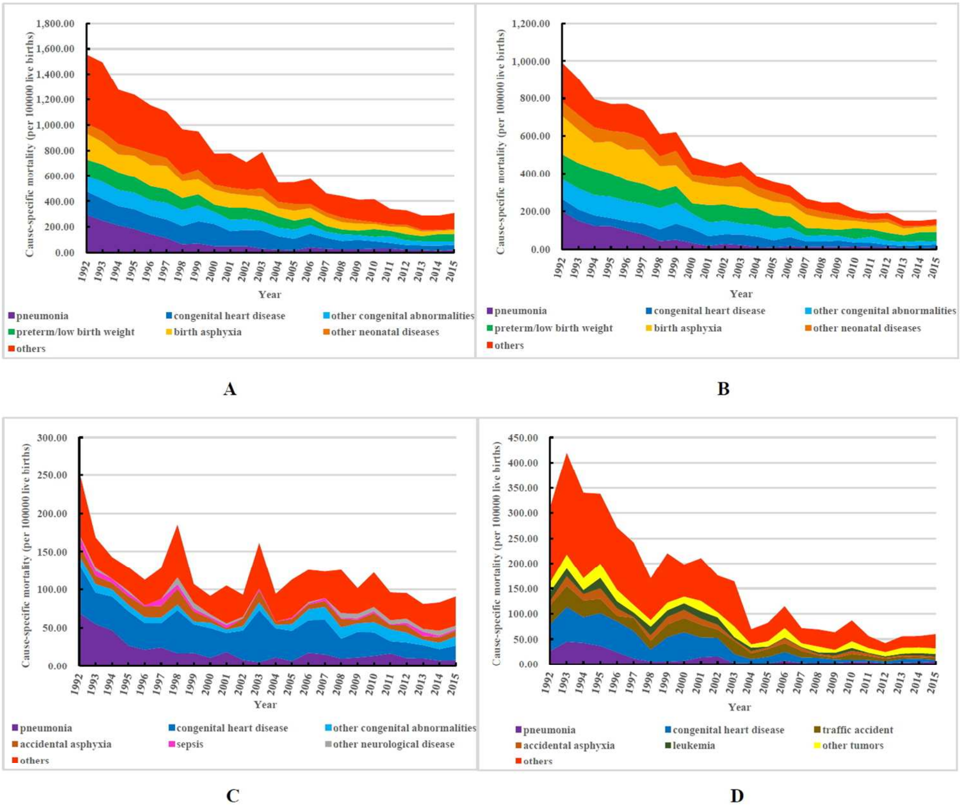

Fig. 2 Trends in cause-specific mortality rates among different ages in Beijing, China, 1992–2015: A. under-5 children;

Fig 2. Trends in cause-specific mortality rates among different ages in Beijing, China, 1992–2015: A. under-5 children; B. neonates (<28 days); C. 28 days to <1 year; D. 1–4 years.

132x116mm (300 x 300 DPI)

only

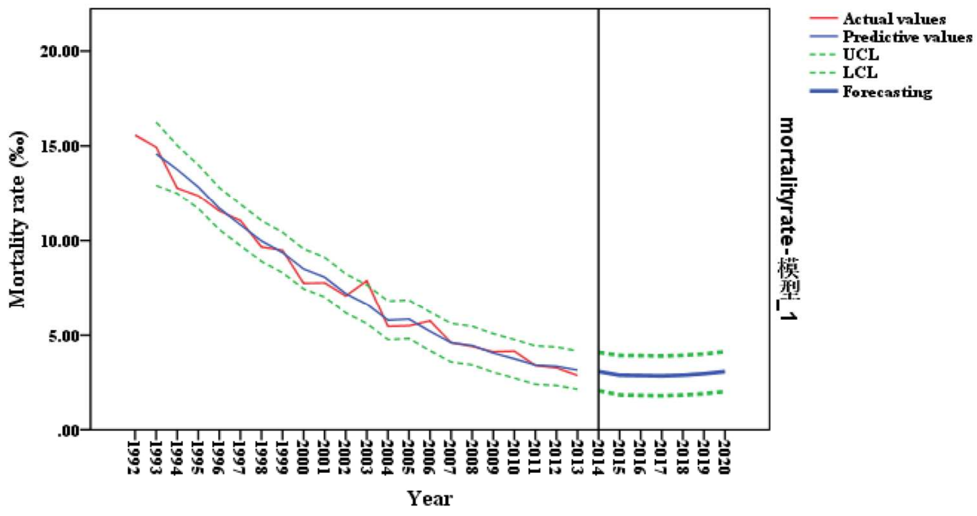

Fig. 3 Prediction of ARIMA (1, 1, 1) model.

(UCL, upper control limit; LCL, lower control limit)

Fig 3. Prediction of ARIMA (1, 1, 1) model (UCL, upper control limit; LCL, lower control limit).

157x99mm (300 x 300 DPI)

STROBE Statement

Checklist of items that should be included in reports of observational studies

Section/Topic	Item No	Recommendation	Reported on Page No
**Title and abstract**	1	(*a*) Indicate the study's design with a commonly used term in the title or the abstract	2
		(*b*) Provide in the abstract an informative and balanced summary of what was done and what was found	2
**Introduction**			
Background/rationale	2	Explain the scientific background and rationale for the investigation being reported	4
Objectives	3	State specific objectives, including any prespecified hypotheses	4
**Methods**			
Study design	4	Present key elements of study design early in the paper	4
Setting	5	Describe the setting, locations, and relevant dates, including periods of recruitment, exposure, follow-up, and data collection	4-5
Participants	6	(*a*) *Cohort study*—Give the eligibility criteria, and the sources and methods of selection of participants. Describe methods of follow-up<br>*Case-control study*—Give the eligibility criteria, and the sources and methods of case ascertainment and control selection. Give the rationale for the choice of cases and controls<br>*Cross-sectional study*—Give the eligibility criteria, and the sources and methods of selection of participants	4-5
		(*b*) *Cohort study*—For matched studies, give matching criteria and number of exposed and unexposed<br>*Case-control study*—For matched studies, give matching criteria and the number of controls per case	This is a cross-sectional study
Variables	7	Clearly define all outcomes, exposures, predictors, potential confounders, and effect modifiers. Give diagnostic criteria, if applicable	5
Data sources/measurement	8*	For each variable of interest, give sources of data and details of methods of assessment (measurement). Describe comparability of assessment methods if there is more than one group	4-5
Bias	9	Describe any efforts to address potential sources of bias	5
Study size	10	Explain how the study size was arrived at	5
Quantitative variables	11	Explain how quantitative variables were handled in the analyses. If applicable, describe which groupings were chosen and why	5-6
Statistical methods	12	(*a*) Describe all statistical methods, including those used to control for confounding	5-6
		(*b*) Describe any methods used to examine subgroups and interactions	5-6
		(*c*) Explain how missing data were addressed	5
		(*d*) *Cohort study*—If applicable, explain how loss to follow-up was addressed<br>*Case-control study*—If applicable, explain how matching of cases and controls was addressed<br>*Cross-sectional study*—If applicable, describe analytical methods taking account of sampling strategy	5
		(*e*) Describe any sensitivity analyses	6

For peer review only - http://bmjopen.bmj.com/site/about/guidelines.xhtml

Section/Topic	Item No	Recommendation	Reported on Page No
**Results**			
Participants	13*	(a) Report numbers of individuals at each stage of study—eg numbers potentially eligible, examined for eligibility, confirmed eligible, included in the study, completing follow-up, and analysed	6
		(b) Give reasons for non-participation at each stage	Entire population-based epidemiological study
		(c) Consider use of a flow diagram	6
Descriptive data	14*	(a) Give characteristics of study participants (eg demographic, clinical, social) and information on exposures and potential confounders	6-8
		(b) Indicate number of participants with missing data for each variable of interest	Entire population-based epidemiological study
		(c) *Cohort study*—Summarise follow-up time (eg, average and total amount)	This is a cross-sectional study
Outcome data	15*	*Cohort study*—Report numbers of outcome events or summary measures over time	This is a cross-sectional study
		Case-control study—Report numbers in each exposure category, or summary measures of exposure	This is a cross-sectional study
		Cross-sectional study—Report numbers of outcome events or summary measures	6-8
Main results	16	(*a*) Give unadjusted estimates and, if applicable, confounder-adjusted estimates and their precision (eg, 95% confidence interval). Make clear which confounders were adjusted for and why they were included	6-8
		(*b*) Report category boundaries when continuous variables were categorized	6-8
		(*c*) If relevant, consider translating estimates of relative risk into absolute risk for a meaningful time period	There is no relevant analysis in this study
Other analyses	17	Report other analyses done—eg analyses of subgroups and interactions, and sensitivity analyses	9-11
**Discussion**			
Key results	18	Summarise key results with reference to study objectives	11-13
Limitations	19	Discuss limitations of the study, taking into account sources of potential bias or imprecision. Discuss both direction and magnitude of any potential bias	14
Interpretation	20	Give a cautious overall interpretation of results considering objectives, limitations, multiplicity of analyses, results from similar studies, and other relevant evidence	14
Generalisability	21	Discuss the generalisability (external validity) of the study results	14

For peer review only - http://bmjopen.bmj.com/site/about/guidelines.xhtml

For peer review only

**Other Information**			
Funding	22	Give the source of funding and the role of the funders for the present study and, if applicable, for the original study on which the present article is based	14

**Give information separately for cases and controls in case-control studies and, if applicable, for exposed and unexposed groups in cohort and cross-sectional studies.*

Note: An Explanation and Elaboration article discusses each checklist item and gives methodological background and published examples of transparent reporting. The STROBE checklist is best used in conjunction with this article (freely available on the Web sites of PLoS Medicine at http://www.plosmedicine.org/, Annals of Internal Medicine at http://www.annals.org/, and Epidemiology at http://www.epidem.com/). Information on the STROBE Initiative is available at www.strobe-statement.org.

For peer review only
